# Non-equilibrium condensation of the first Solar System solids

**DOI:** 10.1038/s41586-026-10257-5

**Published:** 2026-04-22

**Authors:** Sébastien Charnoz, Jérôme Aléon, Marc Chaussidon, Paolo A. Sossi, Yves Marrocchi, Patrick Franco

**Affiliations:** 1 Université Paris Cité, Institut de Physique du Globe de Paris, CNRS, Paris, France; 2 Institut de Minéralogie, de Physique des Matériaux et de Cosmochimie, UMR 7590, Sorbonne Université, Museum National d’Histoire Naturelle, CNRS, Paris, France; 3 Institute of Geochemistry and Petrology, ETH Zürich, Zürich, Switzerland; 4 Université de Lorraine, CRPG, CNRS, UMR 7358, Nancy, France

**Keywords:** Early solar system, Mineralogy

## Abstract

Primitive meteorites (chondrites) consist of an out-of-equilibrium assemblage of minerals formed during the assembly of our solar nebula^1^. The conditions under which their precursors condensed remain unclear as a result of subsequent reprocessing in the protoplanetary disk or in asteroidal parent bodies. Chondrites are classified into three main classes—enstatite, ordinary and carbonaceous—and these are distinguished by different bulk composition and oxidation state^2^. Although equilibrium condensation models explain the composition of some of their refractory components^3,4^, they do not explain the emergence of three mineralogical classes. Moreover, the low pressures, steep temperature gradients and short dynamical transport timescales in forming protoplanetary discs probably hindered equilibrium. Here we test the hypothesis that chondrite precursors formed via kinetic non-equilibrium condensation. Using a new time-dependent condensation model, we show that varying the cooling rate and pressure produce only three types of mineralogies. Departure from equilibrium yields increasingly oxidized and hydrous mineralogies. When projected into a Urey–Craig diagram, the predicted mineralogical types fall close to the redox states of enstatite, ordinary and carbonaceous chondrites. These results suggest that the mineralogical diversity of chondrites may reflect, in part, local condensation kinetics, offering an alternative to large-scale variations of oxidation conditions.

## Abstract (web summary)

A time-dependent model shows chondrite precursors formed by non-equilibrium condensation: cooling rate and pressure alone generate three mineral classes with increasing oxidation, matching enstatite, ordinary and carbonaceous chondrites without large redox variations in the solar nebula.

## Main

The mineralogy of the oldest objects in primitive meteorites, the calcium-aluminium-rich inclusions (CAIs), seems to be successfully explained via an equilibrium condensation sequence (ECS) from a gas of solar composition^4,5^. These calculations are performed assuming that the gas and condensed phase(s) are able to equilibrate with each other at any given temperature and pressure. For temperatures in the 1,300–2,000 K range, the mineralogical sequence found in the ECS is similar to those observed in fluffy CAIs and some amoeboid-olivine aggregates (AOA)^3^. However, as CAI minerals (for example, hibonite, melilite, spinel, grossite, Al–Ti-rich diopside, perovskite) vanish from the ECS below approximately 1,400 K (ref. ^6^), it has long been recognized^4,7,8^ that some degree of disequilibrium is required to explain their coexistence with lower-temperature condensates (for example, olivine, orthopyroxene, Fe–Ni alloy, forming at ≤1,350 K at 10^−4^ bar). Early models invoked fractionated condensation, where solids formed in equilibrium were removed from the gas^9^, leading to the introduction of ad hoc isolation factors^7,8,10^—although it remains unclear what determines their precise value^6^.

In this context, the origin of the broad chemical variations between different classes of chondrites^2^ remains uncertain. Enstatite chondrites possess iron entirely in their reduced (metallic) form, whereas increasing amounts of FeO are present in ordinary and, particularly, carbonaceous chondrites. Although these variations may reflect, in part, secondary processes in their parent bodies, they also trace differences in the mineralogy of the initial condensate precursors^11^. However, ECS models, even when assuming non-solar gas compositions, fail to reproduce the observed range of mineralogical diversity^12^ (Supplementary Sections 1 and 2). An alternative hypothesis states that each class of chondrites formed under different redox conditions^5,13^: silicates in carbonaceous chondrites would have condensed at oxygen fugacities (*f*O_2_) near the iron–wüstite (IW) buffer^14^, whereas the components of enstatite chondrites would have formed under highly reducing conditions. Yet generating such extreme local variations of *f*O_2_ within the solar nebula remains a major problem. The protosolar gas is intrinsically highly reducing (H/O > 2,000, H/C > 3,000), making significant oxidation shifts physically unlikely. Mechanisms such as midplane enrichment in oxygen-rich dust^4,10,15^ or inward transport of carbon-rich gas from the outer disk^14^ have been proposed, but the required levels of enrichment are extremely difficult to achieve under realistic disk conditions^13,15^. As this paradox is based on the premise of equilibrium condensation, here we examine the effect of non-equilibrium condensation, as a natural consequence of low-pressure and vigorous dynamical environments, on the chemical properties of nebular condensates.

## Exploring non-equilibrium condensation

Non-equilibrium condensation is expected in protoplanetary discs. Amoeboid-olivine aggregates in carbonaceous chondrites show strong enrichments in light silicon isotopes, consistent with rapid condensation over approximately 0.01 years under kinetic control^16^. Similarly, light tellurium isotopes in CC chondrules suggest fast, non-equilibrium condensation of chondrule precursors^17^. As reaction rates decrease with temperature and pressure, equilibrium becomes increasingly unlikely at greater distances from the star, where temperatures are lower, or above the disk midplane, where pressure drops exponentially^18^.

The collapse of the molecular cloud onto the solar nebula involves complex dynamics^19–21^, including heating near the star or in accretion shocks, followed by large-scale redistribution across a wide density range. In a minimum-mass solar nebula, pressures range from about 0.1–1 bar inside 0.1 AU to about 10^−5^ to 10^−7^ bar beyond a few AU (Supplementary Section 4). At 1 AU and *P* ≈ 10^−4^ bar, 10 µm grains grow in weeks to months^22^, which is comparable with local free-fall times. Beyond 1 AU, collision rates fall^23^, and equilibration times exceed disk lifetimes—or even the age of the Universe (Supplementary Fig. 12). These constraints motivate study of non-equilibrium kinetic condensation.

**Fig. 1:**
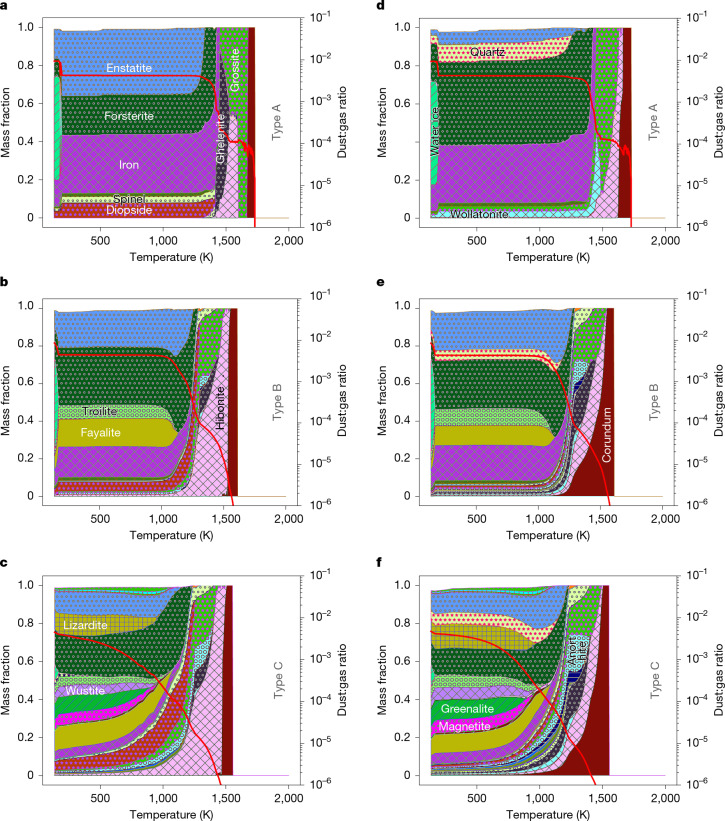
Example of kinetic condensation sequences. **a**–**f**, Resulting mineralogical sequences calculated for FNR (**a**–**c**) and SNR (**d**–**f**) models. Panels show the evolution at *P* = 10^−3^ bar, *T*_c_ = 1,000 years, *X* = 0 (**a**,**d**); *P* = 10^−5^ bar, *T*_c_ = 1 year, *X* = −5 (**b**,**e**); and *P* = 10^−6^ bar, *T*_c_ = 1 year, *X* = −6 (**c**,**f**). Coloured regions indicate the solid mass fraction of each mineral versus temperature; only phases exceeding 1% of total solid mass are shown. The red lines denote the dust:gas mass ratio. Refer to Supplementary Fig. 17 for a colour legend. Mineralogical types A, B and C are discussed in the text. For *T* < 700 K, olivine/(olivine + pyroxene) mass ratios are 32%, 65% and 66% for FNR cases (**a**–**c**), and 81%, 63% and 66% for SNR cases (**d**–**f**).

For this purpose, we have developed a kinetic condensation code KineCond (detailed in the Methods and Supplementary Section 3). It computes the time-dependent condensation and evaporation of a gas of solar composition during cooling from 2,000 K to 130 K at constant pressure *P* and during cooling timescale *T*_c_ in a closed system. Gas–gas reactions are maintained in equilibrium, whereas gas–grain reactions proceed kinetically. The reaction network couples 39 congruant condensation or evaporation reactions (Supplementary Section 3.2 and Supplementary Table 2) and 38 gas–mineral nebular reactions (Supplementary Section 3.4 and Table 3) using an operator-splitting scheme with adaptive time stepping that conserves mass (Supplementary Fig. 5). Minerals grow by direct condensation balanced by evaporation, whereas mineral nebular exchange enables mineral transformations. We explore pressures from 10^−9^ to 10^−2^ bar and cooling timescales from 0.01 to 1,000 years. Due to lack of experimental data we varied nebular reaction rates (activation energies) to simulate fast (FNR), moderate (MNR) and slow nebular reaction (SNR) rates (Methods and Supplementary Section 3.4). Evaporation efficiencies (*γ*) are set to 0.1, in the range of experimental constraints^24^. To compare different condensation conditions, we define an empirical parameter *X* = log_10_(*T*_c_ (year)) + log_10_(*P* (bar)). The minerals formed in representative kinetic condensation sequences (KCS) for −6 ≤ *X* ≤ 0 appear in Fig. 1 (further cases ranging from −11 to +1 appear in Supplementary Section 6).

## Equilibrium versus kinetic regimes

The KCS approximates ECS behaviour under long cooling timescales, high pressure and FNRs (Fig. 1a; *P* = 10^−3^ bar, *T*_c_ = 1,000 years, *X* = 0). From 2,000 K to about 800 K, the KCS closely resembles the ECS (see ECS reference in Supplementary Fig. 1), with calcium- and aluminium-rich minerals condensing first, followed by metallic iron and enstatite forming preferentially over forsterite. In contrast to ECS, KCS mineralogy remains largely unchanged below 800 K due to the exponential decline in reaction rates (Arrhenian kinetics), effectively isolating the condensates from the gas and preserving intact minerals typical of CAIs, even at low temperature. This outcome is achieved naturally in KineCond without invoking an ad hoc isolation factor^7,8,22^. Water ice condenses at about 180 K.

Although ECS and KCS show broad agreement for *X* > –5 and *T* > 800 K, key differences persist. The condensation sequence of high-temperature CAI-relevant minerals varies with nebular reaction rates (FNR versus SNR; Fig. 1a,d), although corundum remains the first condensate. Plagioclase (anorthite, albite) forms in ECS but not in KCS: in ECS, it condenses below approximately 1,400 K (or 1,200 K at 10^−3^ bar), whereas in KCS, calcium and aluminium are already locked into higher-temperature minerals by the time temperatures allow plagioclase formation. This reflects a kinetic barrier absent in ECS, where all elements are assumed to remain available regardless of temperature. By contrast, KCS captures the progressive chemical depletion of the gas under non-equilibrium conditions.

## Formation of three mineralogical types

The general agreement between KCS and ECS at high pressure and long cooling time breaks down when condensation occurs at low pressure or for short cooling time intervals (*X* < −5). Figure 1c,f presents the case at *P* = 10^−6^ bar and *T*_c_ = 1 year (that is, *X* = −6). After the condensation of high-temperature minerals (down to about 1,200 K) and major silicates (1,000 K < *T* < 1,200 K), a rich variety of minerals condense for *T* < 1,000 K. Iron is present in a variety of oxidation states in co-existing phases, including iron metal, fayalite (Fe_2_SiO_4_) and magnetite (Fe_3_O_4_). Phyllosilicates also condense (here, greenalite and lizardite). When cooling is fast enough (*X* ≤ −5), anorthite, melilite (here akermanite and gehlenite) and spinel—three important components found in CAIs—form (Fig. 1b–e).

We have led a systematic exploration of the KCS for different *P* and *T*_c_ (displayed in the forms of mosaics in Supplementary Section 6). Contrary to intuition, KCS does not evolve smoothly with *T*_c_ and *P* (that is, *X*), but instead exhibits sharp transitions, defining only three distinct mineralogical types: types A (Fig. 1a,d), B (Fig. 1b,e) and C (Fig. 1c,f). Type A appears for *X* > −5 (upper-right triangle in matrix-mosaics displayed in Supplementary Section 6), corresponding to high-pressure, slow-cooling conditions and a mineralogy akin to enstatite chondrites (metallic iron and enstatite-rich). At the other extreme, type C occurs for *X* < −5 (lower-left triangle in matrix-mosaics displayed in Supplementary Section 6), corresponding to low-pressure, fast-cooling and characterized by oxidized iron phases (fayalite, magnetite, troilite, phyllosilicates) co-existing with high-temperature CAI minerals (hibonite, grossite) and various silicates. Type B (*X* = −5) is transitional (along diagonals in matrix-mosaic displayed in Supplementary Section 6) with iron condensing as metal, troilite and fayalite from around 1,150 K—which is a higher temperature than in ECS (around 650–500 K). Notably, each type shows limited internal variation despite large changes in *P* and *T*_c_.

Varying the activation energies of nebular reactions by several orders of magnitude has little effect on the resulting mineralogy (compare left and right columns of Fig. 1 and matrix mosaics displayed in Supplementary Section 6). This suggests that phase formation is primarily controlled by the condensation of supersaturated species, with gas–grain reactions playing a secondary role despite the lack of experimental kinetic data (Supplementary Section 3.4.2).

## Oxidizing material in fast cooling processes

**Fig. 2:**
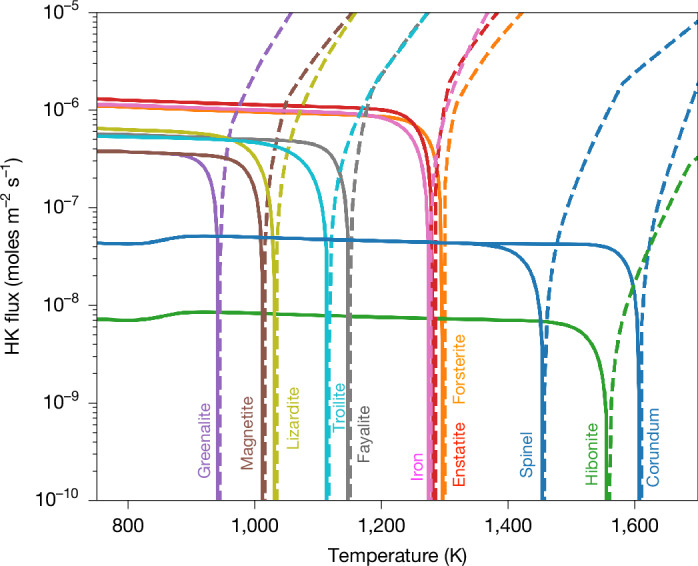
Hertz–Knudsen flux for a selection of minerals. Fluxes are displayed as a function of temperature, in a gas maintaining its solar composition (*P* = 10^−5^ bar). Solid line, condensation flux; dashed line: evaporation flux. A mineral condenses when the evaporation flux drops below the condensation flux, corresponding to super-saturation *S* > 1 (ref. ^46^).

At low pressure and short cooling timescales, mineral diversity results from the temperature-dependent availability of atoms in the gas, governed by condensation kinetics. Disequilibrium arises when cooling outpaces condensation, causing the gas to temporarily retain its high-temperature composition despite the low temperature. In the extreme case in which the gas remains solar at any temperature (as a proxy for ultra-fast cooling), instantaneous condensation and evaporation fluxes are shown (Fig. 2; corresponding supersaturation coefficients are provided in Supplementary Section 7). At about 1,600 K, calcium and aluminium atoms can condense into CAI-like minerals (corundum, hibonite), whereas iron, magnesium and silicon condense into olivine and metallic iron near around 1,300 K at 10^−5^ bar. Full condensation of iron gas atoms takes 0.1–1 year (for 10 µm grains). If cooling is faster, some iron atoms remaining in the gas can later condense as fayalite or troilite near 1,150 K and 1,110 K. In extreme disequilibrium, where significant iron, silicon and magnesium atoms still remain in the gas below 1,050 K, oxidized phases such as magnetite and phyllosilicates (greenalite, lizardite here) condense directly from the gas. This contrasts with earlier models^9^ in which iron is entirely locked in metal, preventing the formation of later iron-bearing phases. In our simulations, the amount of residual gas-phase iron is dictated by condensation kinetics at high temperature (Fig. 2), allowing greater mineral complexity as disequilibrium increases (Fig. 1), particularly for types B and C (*X* ≤ –5). Finally, water ice only forms in types A and B whereas in type C, oxygen is stored in O–H bonds in phyllosilicates rather than as crystalline H_2_O ice (Fig. 1a,b,d,e).

## Condensation paths in the Urey–Craig diagram

**Fig. 3:**
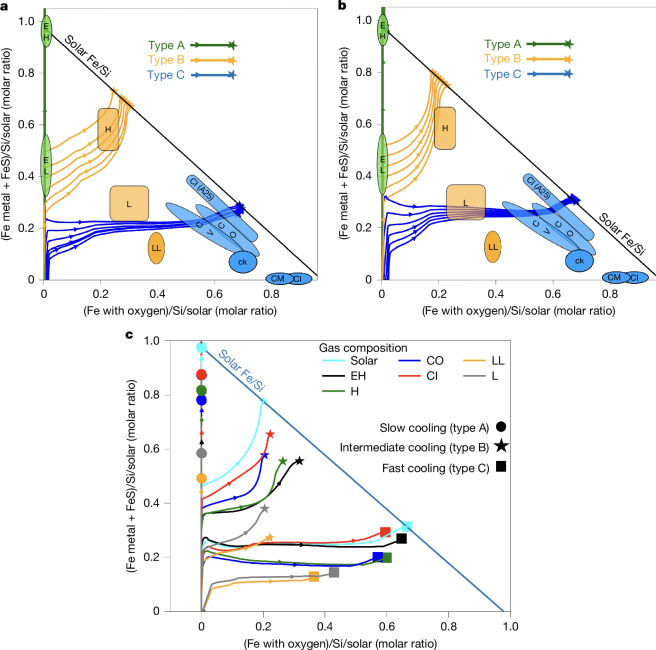
Evolution of condensates in the Urey–Craig diagram. This diagram shows the iron redox state of condensates along the condensation path calculated with KineCond, compared with chondrite compositions (coloured shapes). The *x*-axis shows molar fraction of oxidized iron, whereas the *y*-axis shows iron in metal and sulfide, normalized to silicon and to the solar Fe/Si ratio. Coloured solid lines trace the redox evolution during condensation process of types A, B and C for various (*P*, *T*_c_) cooling conditions. **a**, Condensation paths of a solar-composition gas with FNRs. **b**, Same as **a**, but for SNRs. All trajectories end on the solar iron–silicon line. **c**, Condensation paths for non-solar gas compositions, using moderately fast reactions. Colours correspond to different gas compositions and marker shapes denote different cooling rates (*X* = −6, −5, −2).

We use the three chondrite classes—EC, OC and CC—as a conceptual framework for exploring redox evolution and bulk mineralogy. Although this simplification overlooks the full diversity of the sixteen recognized chondrite groups (such as the distinction between H, L and LL chondrites), it aims to elucidate differences in precursor material rather than reproduce the exact properties of each subgroup. The evolution of iron oxidation in our condensates is shown in the Urey–Craig diagram (solid lines in Fig. 3a,b). All condensation sequences start in the lower-left corner and end on the solar iron/silicon line once condensation reaches 100% (reflecting our assumption of closed solar-composition system). Despite wide variations in pressure and cooling times (−6 < *X* < 0), final condensates cluster into three regions along this line, qualitatively matching the redox states of EC, OC and CC chondrites (Fig. 3a,b). Type A mineralogies contain no oxidized iron and are dominated by enstatite over olivine and sulfides (*<*1 wt%), qualitatively consistent with EH chondrites. Type B paths starts near the EL field and progressively incorporate iron into fayalite. The fast-reaction cases end near H chondrites when condensation is complete, whereas slow reaction cases retain more metallic iron. Type C trajectories are more complex: they pass near L–LL fields, then through CO–CV regions, and end very oxidized, with iron mostly in fayalite, magnetite, troilite and phyllosilicates (greenalite). Although their final states are less oxidized than classical CM or CI chondrite values, they are very close to the revised CI estimate (A25 in Fig. 3)^25^. Higher oxidation states (*>*0.7) are not reproduced by KineCond and probably result from parentbody alteration^26^. As chondrite groups also exhibit non-solar bulk compositions (which origin is not invesigated here), we next explore how varying gas composition affects condensation paths using non-solar mixtures reflecting Fe/Si, Mg/Si and Al/Si measured in different chondritic sub-groups (Supplementary Table 1). Varying gas composition modifies the final oxidation state of the condensates (Fig. 3c), but all trajectories remain clustered in the Urey–Craig diagram. Slow condensation always produces reduced type A assemblages, whereas rapid cooling yields oxidized type C assemblages—independent of bulk gas composition. Condensation of L and LL-like gas compositions at *X* = − 5 end closer to the observed L and LL fields but still remain systematically too reduced (see orange and grey star markers in Fig. 3c). Thus, the fact that our model captures only the three chondrite classes (EC, OC, CC) at first order in the Urey–Craig diagram, but not their subgroups, may be due to missing physics (that is, silicate or metal sorting due to aerodynamic drag, C or O variations at the disk scale, open-system, gas–solid separation or parent-body alteration), indicating that kinetic condensation alone cannot explain the full diversity of chondrites.

## Apparent *f*O_2_ of kinetic condensates

The Urey–Craig diagram, by itself, does not reflect the *f*O_2_ of the environment in which the chondrites formed because the components of primitive chondrites are unequilibrated.

Estimates for the reequilibrated *f*O_2_ of chondrites have been made using different techniques^27,28^.

Enstatite chondrites record nominal *f*O_2_ values consistent with that of a gas of solar composition (about ∆IW ≈ −6 to −7). CI chondrites are more oxidized (with IW in the range of −4 to 0) than expected during condensation of a solar gas, unless temperatures fall below 400 K (ref. ^27^). The leading hypothesis for their oxidation is the circulation of aqueous fluids on their parent bodies^26^.

To estimate the apparent *f*O_2_ of the non-equilibrium mineral assemblages A, B and C, the corresponding equilibrium *f*O_2_ was calculated at *T* = 1,500 K and *P* = 10^−4^ bar using the Factsage software (Supplementary Section 8 and Supplementary Figs. 19 and 20). Types A, B, C show re-equilibrated bulk compositions with distinct *f*O_2_ trajectories as a function of temperature. When all condensable material is in solid form, types A, B and C show re-equilibrated *f*O_2_ of ∆IW ≈ 4, ∆IW ≈ −2 and ∆IW ≈ −0.5, respectively, spanning the range of estimated *f*O_2_ in the different classes of (equilibrated) chondrites^14,28^. These equivalent *f*O_2_ values deviate from the true *f*O_2_ values at which these minerals condensed (the gas remains at ∆IW ≈ −6 to −7 over most of the temperature range; Supplementary Fig. 20). Type C mineralogies are especially oxidized, and also have a water mass fraction of about 1–2 wt%, without appealing to addition (that is, super-solar) of oxygen or water to the system. High-temperature minerals, condensed at *T* > 1,200 K in KineCond, still have −6 *< *∆IW < −10, at low temperature, close to the true *f*O_2_ of their formation environment.

## From precursors to the chondrite components

Although KineCond redox trends broadly align with the three chondrite classes, whether kinetic condensation also shaped specific components, particularly chondrules, remains uncertain. These underwent significant reprocessing: melting, recycling, gas–grain interactions and aqueous alteration, before their incorporation into meteorites^29^. Nevertheless, some chondrule mineralogies remain qualitatively consistent with a kinetic condensation origin.

Ferro-magnesian chondrules, the most abundant type, are classified on the basis of their olivine:topyroxene ratio (above 80% olivine for type A, 20–80% for type AB, and below 20% for type B) and iron oxidation state (FeO *< *5 wt% for type I; FeO *>* 5 wt% for type II)^30^. Type I chondrules typically contain abundant iron metal and are thought to form under reducing conditions. In KineCond, slowly cooled type A condensates (*X* ≃ 0) yield forsterite-rich mineralogies with iron metal—similar to AOAs and other type IA precursors^31,32^.

Faster-cooling type B condensates yield more oxidized assemblages with fayalitic olivine and residual metal, broadly resembling type II precursors. This suggests that oxidation could result from disequilibrium condensation rather than the addition of external oxidants (for example, CI-like dust or ice)^12^. Although KineCond does not resolve the full chemical complexity of chondrules, it suggests a coherent framework where redox diversity arises from local variations in pressure and cooling rate. The model also predicts co-existence of iron metal, FeS and oxidized phases (Fig. 1 and Supplementary Fig. 6), consistent with mineral assemblages in chondrules^23,33^. Simulations of transient heating events, such as in bow shocks^34^, followed by recondensation always converge to one of the three end-member mineralogies, depending on the cooling rate (Supplementary Section 10). Matrix materials probably reflect both condensation and parent-body alteration; however, primary amorphous silicates—thought to be initial matrix phases—cannot currently be modelled due to missing thermodynamic data^26^, and their mineralogy has been extensively modified by thermal and aqueous processes^35^.

## Astrophysical context

**Fig. 4:**
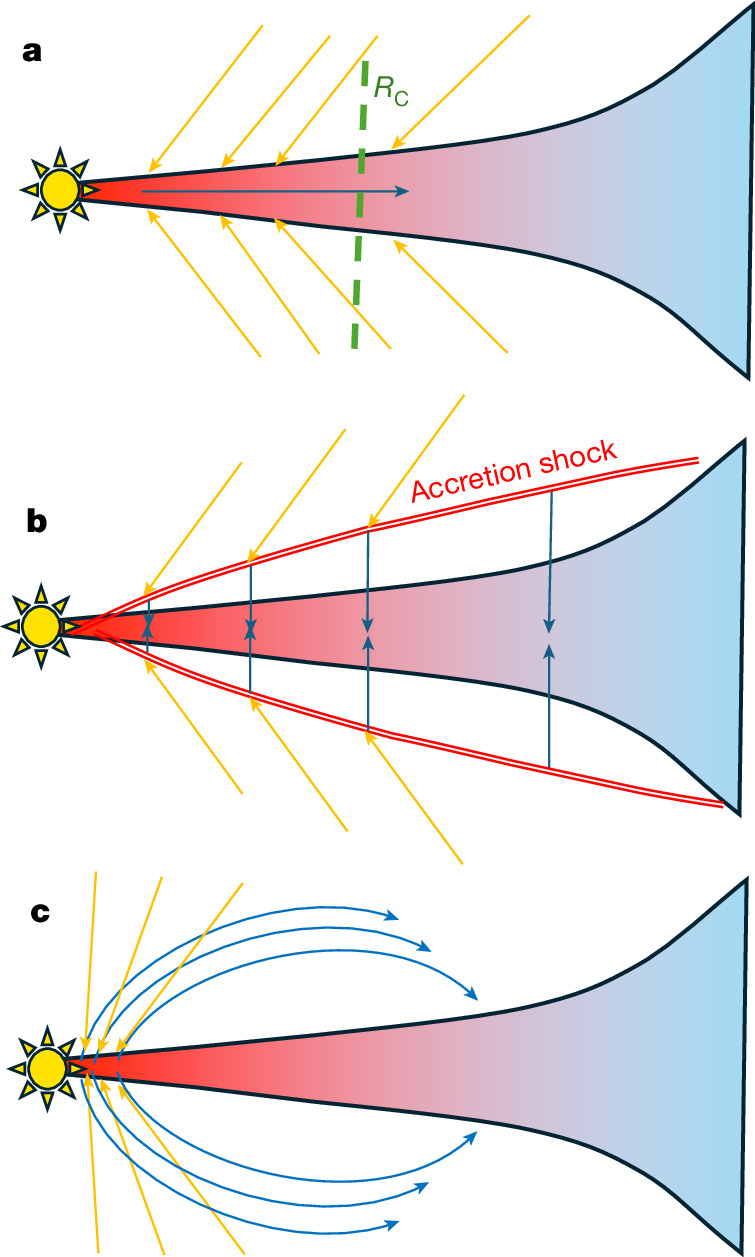
Possible accretion pathways of molecular-cloud gas and redistribution. Three schematic scenarios for the inflow of ISM gas into the forming solar nebula. Orange arrows indicate heated gas, whereas blue arrows indicate cooling gas. The red–blue ellipse represents the disk, with red denoting hot, high-pressure regions (*>*2,000 K, *>*10^−3^ bar) and blue denoting cold, low-pressure regions (*<*1,000 K, *<*10^−4^ bar). **a**, Gas accretes inside the centrifugal radius (*R*_c_), condenses near the star at high temperature and pressure, and is redistributed by viscous spreading. **b**, Infalling gas passes through a hot accretion shock, cools and condenses before entering the disk. **c**, Hot gas is injected near the star and is ejected outward, with condensation occurring during cooling in the outflow.

Were solar nebula conditions suitable for producing type A, B and C mineralogies? This depends on how interstellar gas accreted onto the protoplanetary disk—a process still debated. Figure 4 outlines three proposed scenarios. In one-dimensional collapse models conserving angular momentum^36–39^, gas accretes near the proto-Sun where pressure and temperature are highest (see Supplementary Section 4), favouring type A mineralogies close to the star and type C farther out; however, slow viscous spreading^38,39^ limits cooling rates, probably preventing significant non-equilibrium effects. More recent two- and three-dimensional simulations^19–21,40^ reveal infall extending out to around 10 AU in asymmetric patterns. Accretion shocks above the midplane reach *T* > 2,000 K (refs. ^20,21^), allowing thermal processing before gas cools and condenses into type A, B or C mineralogies (Fig. 4b) in the midplane. Furthermore, outflows near the proto-star may eject hot material that cools rapidly in low pressure environment, promoting non-equilibrium condensation (Fig. 4c).

We propose that mineral precursors condensed under the conditions described here, and that their initial mineralogy influenced the outcome of subsequent disk and asteroidal evolution processes.

Observations of class I protoplanetary discs reveal radial gaps that can act as dust barriers and limit radial mixing^41–43^. In our Solar System, the early spatial and temporal separation of NC and CC reservoirs supports this idea^44,45^. Such separation may have helped preserve the distinct mineralogies and bulk compositions of the precursors that gave rise to the three chondrite classes.

Although based on simplifying assumptions—due to computational limits and scarce experimental data (Methods)—our model suggests that non-equilibrium condensation may have contributed to the redox diversity of precursors and the first-order division of chondrites. These kinetic effects probably operated alongside other processes not included here, such as C/O variations, gas–solid separation, physical sorting and parent-body alteration. Given that chondrite classes also differ in bulk composition, which our closed-system approach does not capture, our framework should be viewed as a conceptual basis rather than a full explanation.

## Methods

KineCond is a code developed to calculate the time-dependent condensation processes of minerals in the solar nebula, which is dominated by hydrogen. It is described in detail in Supplementary Section 3 with several validation tests, and here we give a summarized version. The elements considered in the system are H, He,O, Mg, Si, Fe, Al, Na, K, Ni, Ca, Cr, S and C. Here, the term element designates an atomic species. The system consists of a gas in interaction with 39 minerals. Most of the condensation codes published in the past and that allowed the investigation of the Equilibrium Condensation Sequence^3–5,7^ rely on the Gibbs free-energy minimization (GFEM). GFEM calculates—for a given pressure and temperature—the most stable combination of minerals and gas; however, like any equilibrium calculation, it provides no information if the equilibrium state is established in a reasonable time, nor does it give the list of reactions through which the equilibrium state is realized (even though some additional physical arguments may help to determine those reactions, especially at high temperature when the number of minerals present is small). However, each reaction has its own kinetics, which depends on pressure, temperature and the local abundances of all elements. Thus, to design a time-dependent kinetic code, we must adopt a different strategy. We must explicitly specify the list of all reactions of interest, and advance each of them individually in a fully coupled way.

KineCond proceeds as follows: at each time step, we compute the number of atoms in the gas and the number of atoms in every mineral, keeping the total number of atoms constant. We assume that the pressure is constant and that only the temperature varies with time. The evolving variables of the system are: the number of moles of each molecule *i* in the gas (*N*_*i*_^gas^) and the number of moles of each mineral *j* in the system (*N*_*j*_^min^; Supplementary Table 2). We assume that gas–gas reactions are much faster than gas–mineral reactions and condensation reactions, so the gas molecular composition is always close to chemical equilibrium (then the gas molecular composition only depends on the current values of temperature and pressure, as well as *N*_*i*_^gas^). As we focus on gas–mineral processes, reactions are divided into two broad categories: (1) condensation or evaporation reactions; and (2) gas–mineral reactions. The rates of these reactions dictate the evolution of *N*_*i*_^gas^ and *N*_*j*_^min^. The temperature varies linearly with time, dropping from 2,000 K to 130 K on the timescale *T*_c_ (ranging from 0.01 to 2000 years). The system evolves as follows: at each time *t* we first calculate the molecular composition of the gas (excluding mineral condensation) by computing the chemical equilibrium of the gas at *T*(*t*) and *P* and with elemental abundances *N*_*i*_^gas^(*t*). This is performed using the iconic Chemical Equilibrium with Application distributed by NASA (CEA-NASA) code^47^, which includes about 1,500 gas species in total. The instantaneous gas molecular composition is then used to compute the different condensation and gas–mineral reactions and the rate at which they proceed. The different steps of the calculation are summarized in a flow chart provided in Supplementary Fig. 5. We detail these calculations below.

Condensation or evaporation reactions We follow a condensation and evaporation theory developed for forsterite evaporation in a H_2_ gas^48,49^, and we generalize it to many minerals. The net formation rate of a mineral *j* is the difference between an evaporation flux (*J*_*j*_^e^) and a condensation flux (*J*_*j*_^c^). Each of them must be computed explicitly. In the gas, the flux of any element *E* across a unit surface (in moles m^–2^ s^–1^) is calculated using the kinetic theory of gases^48,49^:

1 $${J}^{c}(E)=\sum _{m}\frac{{\nu }_{m}^{E}{P}_{m}}{({2{\rm{\pi }}{\mu }_{{\rm{m}}}RT)}^{1/2}}$$

where *m* is any gas molecule; ${\nu }_{m}^{E}$ is the stoichiometric coefficient of element *E* in molecule *m*; *P*_*m*_ is the partial pressure of molecule *m*; *µ*_m_ the molar mass; and *R* is the ideal gas constant. The partial pressures of gas molecules (*P*_*m*_) are obtained by running the CEA-NASA code. We now consider a mineral *j* with formula $\{{\alpha }_{j}^{E}E\}$, where ${\alpha }_{j}^{E}$ is the stoichiometric coefficient of element *E* in mineral *j*. The condensation flux of the mineral *j* (*J*_*j*_^c^) is determined by the smallest flux (over all elements *E* entering in its composition) so that:

2 $${J}_{j}^{{\rm{c}}}={\gamma }_{E,j}{\min }_{E}\left(\frac{{J}^{{\rm{c}}}(E)}{{\alpha }_{j}^{E}}\right)$$

*γ*_*E,j*_ is the sticking efficiency of atom *E* on mineral *j*. It ranges from 0 to 1 and is poorly constrained.

Here we set *γ*_*E,j*_ = *γ* = 0.1, as a standard value, for all minerals and all atomic elements.

The evaporation flux of the mineral *j* (*J*_*j*_^c^) is classically^50^:

3 $${J}_{j}^{{\rm{e}}}=\frac{{P}_{j}^{\mathrm{sat}}}{({2{\rm{\pi }}{\mu }_{j}{RT})}^{1/2}}$$

where *P*_*j*_^sat^ is the saturating vapour pressure of mineral *j* at temperature *T*; *P*_*j*_^sat^ is easily defined in vacuum and for a mineral that has an equivalent gas form with same formula (like H_2_O or Fe).

However, most minerals do not have a corresponding gas form (like Mg_2_SiO_4_), making equation (3) not directly applicable to most minerals. Furthermore, the presence of the surrounding gas (mainly hydrogen and helium) modifies the chemical equilibrium and must be taken into account when computing the saturation vapour pressure. So, equation (3) is not directly applicable to compute the evaporation flux of all minerals immersed in a surrounding gas rich in H. To compute *J*_*j*_^e^ from the kinetic theory of gases, we follow a strategy by which the evaporating flux is directly computed without the need to compute the saturating vapour pressure^48^^,^^49^. When a mineral is in equilibrium with its surrounding gas, the evaporating and condensation fluxes exactly balance (equations (2) and (3)).

So, determining the evaporation flux of the mineral *j* is equivalent to determining the condensation flux of the mineral *j* in equilibrium with the surrounding gas at pressure *P* and temperature *T*. Following previous work^48^ focusing on forsterite equilibrium with H_2_ we have calculated the vapour composition at equilibrium with a mineral immersed in H_2_ gas and derived the evaporative flux using equation (2). This calculation was performed using the CEA code for 39 minerals (Supplementary Table 2). The pressure of the H_2_ gas varied in the 10^−10^ bar *< P* < 0.1 bar range, the temperature *T* was varied in the 130 K < *T* < 2,200 K range, and *f*O_2_ was varied by ten orders of magnitude. All of these data are compiled into lookup tables and are interpolated at current (*T*, *P*, *f*O_2_). So in KineCond the ideal evaporative flux (*F*_*j*_) from the mineral *j* is obtained by reading a precalculated lookup table and interpolated between tabulated values at current values of ln(*P*), *T* and ln(*f*O_2_). Examples of computed evaporative fluxes are shown in Supplementary Fig. 10. The result is multiplied by *γ* = 0.1 so the effective evaporative flux is:

4 $${J}_{j}^{{\rm{e}}}=\gamma {F}_{j}(P,T,f{{\rm{O}}}_{2})$$

The time derivative of number of moles of any mineral *j* due to competing condensation and evaporation processes is then:

5 $$\frac{d{N}_{j}^{\min }}{{dt}}=S({J}_{j}^{c}-{J}_{j}^{e})$$

Where *S* is the surface of contact of the grain with the gas; *J*_*j*_^c^ and *J*_*j*_^e^ are given by equations (2) and (4), respectively. Our code implements a first-order time solver (Euler), and during a time step *dt*, the number of moles of every mineral evolves according to equation (5). To conserve the total number of moles of each element, the atoms released and removed from the gas (*N*_*i*_^gas^) are counted according to the stoichiometry of every mineral. We adopt an operator-splitting approach in which condensation reactions during the *dt* time step are treated first (Section 3.2 of Supplementary Information), and gas-mineral reactions are treated in a second step (Section 3.3 of Supplementary Information).

The surface of contact of minerals with the gas (*S*) depends on the radius of the grain (*r*) and the number of grains (*N*) so that *S* ≈ *N*4π*r*^2^. The self-consistent calculation of *N* and *r* necessitates the computation of the time-dependent nucleation process and that both the sticking and fragmentation processes of minerals during their settling and growth in the turbulent solar nebula are taken into account. So far, such a coupling (mineral condensation, coagulation and fragmentation) has never been realized, and some models take some processes into account (coagulation or fragmentation^51,52^, or metal condensation^22^). Models coupling dust settling, coagulation and fragmentation show that dust size distribution reaches rapidly steady-state inward 10 AU in a few orbital periods^51,53^, and that which determines dust size is the equilibrium between coagulation and fragmentation in turbulence, rather than mineral growth. Following these lines, and to make the calculation tractable, we have used a characteristic dust size, which gives a gas–mineral surface of contact, rather than computing self-consistently a mineral growth model, and we limit ourselves to an order-of-magnitude calculation. In other terms, we assume that all minerals (despite their mass) have a surface in contact with the gas equivalent to a sphere with radius *r* = 10 µm (where *r* is a free parameter of the model). This size is typical of the minerals observed in chondrites. The number of grains in the system (*N*) should be controlled by the number of nucleation sites that first appear in the gas. As aluminium is the most refractory atom in our model, we approximated *N* ≈ *M*_Al_*/*(4*/*3π*ρ*_Al_*r*^3^) where *M*_Al_ is the total mass of aluminium in our system and *ρ*_Al_ is the density of aluminium, so *S* = 3*M*_Al_*/*(*ρ*_Al_*r*). This does not mean that all minerals are 10 μm in radius, but rather that on average the total surface of contact of a mineral is equivalent to a population of minerals with an average surface of a sphere with a 10-µm radius (it could be fractal in shape). Of course, our calculation may not be accurate at the beginning of coagulation when the particle size is close to the monomer size, but changing the monomer size by a factor of 1,000 only changes by 4% the growth time and does not change the final size^22^. Particles with a radius of 70-μm were formed in about ten weeks^8^ (with a cooling rate of about 100 K per year at 10^−4^ bar; a pressure typical of the 0.1–1 AU region), which is comparable to the orbital period at the distance of Mercury. Other works find the formation of 10-μm grains in a few orbital periods at 1 AU (refs. ^51,53^) or at 5 AU (ref. ^52^). Of course, larger minerals can be formed, but 10 μm is typical of what is observed in meteorites.

### Gas–mineral surface reactions (nebular reactions)

In addition to condensation and evaporation reactions, condensed minerals can also interact with the gas, leading to mineral transformation. Nebular reactions are those where the gas interacts with a pre-existing mineral (M1) and forms a new mineral (M2) and consuming M1. The number of such reactions is potentially infinite and for now KineCond implements reactions with the following generic form:

6 $${\rm{M}}1+{\beta }_{1}{\rm{E}}1+{\beta }_{3}{\rm{E}}2\Rightarrow \alpha {\rm{M}}2$$

Where M1 and M2 represent two minerals; E1 and E2 represent any element in gas form; and *α*, *β*_1_ and *β*_2_ are stoichiometric coefficients (normalized so that the stoichiometric coefficient of M1 is 1). For example, Mg_2_SiO_4_(s) + 1 Si + 2O ⇒ MgSiO_3_ (s).

Elements E1 and E2 can be implied in the reaction in any molecular form (silicon could be in the molecular form SiO, Si, SiO_2_ and so on), but the radical of the molecule that is not used in the reaction is released into the gas and does not enter into the calculation of the reaction constant (due to the difference of the formation ∆*G* between the left and right sides of the equation; see Supplementary Sections 3.4 and 3.4.1 for a detailed calculation). For now, we are limited to reactions in which mineral M1 does not lose atoms to the gas. If M1 did, this could be an incongruent evaporation process, and this nebular reaction would be inconsistent with our hypothesis of treating condensation or evaporation as congruent processes using the Hertz–Knudsen formalism described above. Using combinatorial analysis, we found that 55 reactions of this type are possible with our mineral selections (Supplementary Table 2). After many tests, only 38 reactions were kept, the others playing a more minor or no role (Supplementary Table 3).

For a given temperature and for a given gas composition, we first compute whether any reaction listed in Supplementary Table 3 is kinetically possible, that is, if the mineral M2 is formed (see Supplementary Section 3.4 for a detailed calculation). If the reaction favours the formation of the mineral M2, then we calculate the rate at which M2 is formed and M1 disappears.

#### Rate of nebular reactions

We use the simple collision theory^23^ model to compute the reaction rate of reaction 6, but modified the model to take into account improvements in our understanding of reaction rates on the surface of the mineral^24^. We first determine the flux of incoming elements E1 and E2 by summing the fluxes of all molecules in the gas, which carry elements E1 or E2 (equation (1)) called *J*^c^(E1) and *J*^c^(E2), where c is condensation. We call them elementary fluxes. The smallest of the two fluxes (weighted by *β*_1_ or *β*_2_) controls the rate of progression of the reactions. So if reaction 6 is thermodynamically possible, then the rate at which mineral M1 appears (and mineral M2 disappears) is:

7 $$\frac{d{N}_{{\rm{M}}1}^{\min }}{{dt}}=-S\times \min [{J}^{{\rm{c}}}(E1)/{\beta }_{1},{J}^{{\rm{c}}}(E2)/{\beta }_{2}]$$

8 $$\frac{d{N}_{{\rm{M}}2}^{\min }}{{dt}}=-\alpha \frac{d{N}_{{\rm{M}}1}^{\min }}{{dt}}$$

Equation (7) assumes that all collisions lead to the reaction and overestimates the reaction rate; it must therefore be corrected. Laboratory experiments show that two regimes of reaction rates exist^24,33,54,55^: the linear and parabolic regimes. In the former, each molecular collision has a certain probability of producing a chemical reaction, parameterized by the activation energy ${E}_{a}^{l}$. So the linear rate is:

9 $${\left.\frac{d{N}_{{\rm{M}}1}^{\min }}{{dt}}\right|}_{\mathrm{linear}}=-S\times \min [{J}^{{\rm{c}}}(E1)/{\beta }_{1},{J}^{{\rm{c}}}(E2)/{\beta }_{2}]{e}^{-{E}_{{\rm{a}}}^{l}/{RT}}$$

We recover here the simple collision theory model^23^. However, laboratory experiments show that after a first period during which the reaction rim grows linearly with time at the mineral’s surface^33,54^, the reaction switches to a parabolic regime where atomic diffusion across the reactive rim limits the reaction rate. In this regime, the rim grows with the square root of time. In that case, the reactive layer with thickness *H* grows like *H*^2^ = *k*(*T*)*t* where *k*(*T*) is a diffusion coefficient that depends on temperature (m^2^ s^–1^) and *t* is time^33^. It is usual to write $K(T)=C{e}^{-{E}_{a}p/RT}$, where *C* and ${E}_{{\rm{a}}}^{p}$ represent a prefactor and the activation energy (in the parabolic regime), respectively. This process can be described by a Fick diffusion law where the reaction rate (in mol m^−2^ s^−1^) is

10 $$\frac{{dN}}{{dt}}=-{Sk}(T)\frac{{dc}(x)}{{dx}}$$

where *c*(*x*) is the concentration of mineral M2 at location *x* (*x* = 0 corresponds to the surface of mineral M1). To simplify the calculation, we assume that *c*(*x*) drops linearly within M1, so that *dc/dx* ≈ 1*/H*. So we solve:

11 $$\frac{{dN}}{{dt}}=-{Sk}(T)\frac{1}{H}$$

where *H* is the thickness of the mineral M2 layer above the M1 surface and S the grain surface. In KineCond H is calculated as *H* = *µ*_M2_*N*_M2_*/*(*Sρ*_M2_) where *µ*_M2_ and *ρ*_M2_ are the molar mass and density of mineral 2. To avoid non-physical high reaction rates when H is close to 0 we bound the parabolic rate to be always smaller than the rate of incoming atoms to the M1 mineral’s surface.

So, the reaction rate in the parabolic regime reads:

12 $${\left.\frac{d{N}_{{\rm{M}}1}^{\min }}{{dt}}\right|}_{\mathrm{parabolic}}=-S\times \min \left[\frac{k(T)}{H};\,\min [{J}^{c}({\rm{E}}1)/{\beta }_{1},{J}^{c}({\rm{E}}2)/{\beta }_{2}]{e}^{-{E}_{{\rm{a}}}^{l}/{RT}}\right]$$

Unfortunately, there are only a handful of laboratory measurements^24^ and most gas–mineral reactions are undocumented. For magnetite formation, laboratory experiments give ${E}_{{\rm{a}}}^{p}\,\approx \,90\,\mathrm{KJ}\,{\mathrm{mol}}^{\mbox{--}1}$ (ref. ^54^), whereas for Troilite (FeS) the activation energy in the parabolic rate is reported as $30\,\mathrm{kJ}\,{\mathrm{mol}}^{\mbox{--}1} < {E}_{{\rm{a}}}^{p} < 70\,\mathrm{kJ}\,{\mathrm{mol}}^{\mbox{--}1}$ and in the linear regime is $28\,\mathrm{kJ}\,{\mathrm{mol}}^{\mbox{--}1} < {E}_{{\rm{a}}}^{{\rm{l}}} < 94\,\mathrm{kJ}\,{\mathrm{mol}}^{\mbox{--}1}$ (ref. ^33^). These measurements were done in the (*T*, *P*) range of stability of both minerals. In contrast, the rate of forsterite to Enstatite (Mg_2_SiO_4_ + 1Si + 2O → 2MgSiO_3_) was measured at a temperature well above the stability of Enstatite or Forsterite (*>*1,700 K) and *E*_a_^*p*^ ≈ 500 kJ mol^–1^ was found^56^. For magnetite, troilite and enstatite activation energies, we use the laboratory values for the parabolic regime. For the linear regime and for all other reactions due to many uncertainties and lack of data^24^, we investigate different end-member scenarios defined below:

- FNR: (end member) *E*_a_^*l*^ = 0 kJ mol^–1^ and *E*_a_^*p*^ = 0 kJ mol^–1^
- MNR: *E*_a_^*l*^ = 20 kJ mol^–1^ and *E*_a_^*p*^ = 20 kJ mol^–1^
- SNR: *E*_a_^*l*^ = 80 kJ mol^–1^ and *E*_a_^*p*^ = 500 kJ mol^–1^.

The prefactor coefficient in *k*(*T*), *C*, is determined so that the linear and parabolic fluxes connect smoothly for a rim thickness of *H* = 1 µm. This also prevents unrealistically high reaction rates for small values of H (a well-known problem of Fick’s law). So *C* is equal to the linear rate divided by 1 µm, that is, in the range of transition rim thicknesses measured for troilite formation^33^. For the enstatite formation experiment, the transition thickness is below 10 μm (ref. ^56^).

Of course, the above procedure does not reflect the vast richness of gas–grain reactions and suffers from an important lack of experimental data. However, we have performed numerous tests, varying the values of ${E}_{{\rm{a}}}^{l}$ and *E*_a_^*p*^. We found that changing these values does not significantly impact our results as the processes investigated here are dominated by condensation processes, rather than gas-mineral surface interactions.

Putting all things together, the user first specifies the gas composition (solar in general), pressure and cooling time (in years). Temperature will decrease linearly from 2,000 K to 130 K in time *T*_c_. The equivalent cooling rate is therefore (2,000 − 130)*/T*_c_ (K per year). Each time step is decomposed as follows.

1. Compute the gas molecular composition at a given temperature and pressure (using only atoms present in gas form) using the CEA-NASA equilibrium code^47^.
2. Compute the resulting gas atomic fluxes (equation (1)).
3. Condensation/evaporation phase: calculate the condensation and evaporation fluxes for every mineral (equation(5)), and evolve the number of minerals accordingly and the number of elements in the gas.
4. Nebular reactions phase: Determine which nebular reactions occur in the gas (Supplementary Table 3) and compute the rate of production of mineral M2 and the rate of destruction of mineral M1 (equations (9) and (12)).
5. Update the number of elements in the gas, enforcing mass conservation.
6. Increment time and go back to the first step.

Several comparisons of KineCond outputs against various laboratory experiments and a comparison with the classical equilibrium condensation sequences obtained by GFEM are shown at the end of Supplementary Section 3. We find good, to very good, agreement for all these tests.

## Online content

Any methods, additional references, Nature Portfolio reporting summaries, source data, extended data, supplementary information, acknowledgements, peer review information; details of author contributions and competing interests; and statements of data and code availability are available at 10.1038/s41586-026-10257-5.

## Supplementary information

Supplementary Information

## Data Availability

The data used to produce Figs. 1–3 were generated by the KineCond code, available in the IPGP research Collection public repository at 10.18715/IPGP.2026.mkv2scjh.

## Code availability

A version of the KineCond code, configurated to reproduce the results of this paper, is available in the public repository IPGP Research Collection at 10.18715/IPGP.2026.mkv2scjh.
