# Supplementary material for: Non-equilibrium condensation of the first Solar System solids
Source: Nature. 2026 Apr 22;652(8111):925–30. doi: 10.1038/s41586-026-10257-5 (PMC13102690; doi:10.1038/s41586-026-10257-5)
Supplement: Supplementary file 1 — Supplementary Information [file 41586_2026_10257_MOESM1_ESM.pdf]

nature portfolio
https://doi.org/10.1038/s41586-026-10257-5

Supplementary information

Non-equilibrium condensation of the first Solar System solids

In the format provided by the authors and unedited

Supplementary Information: Non-equilibrium condensation of the first Solar System solids

Sébastien Charnoz[1*], Jérôme Aléon[2], Marc Chaussidon[1], Paolo A. Sossi[3], Yves Marrocchi[4] and Patrick Franco[1]

[1*]Université Paris Cité, Institut de physique du globe de Paris, CNRS, 1, rue Jussieu, Paris, F-75005, France.
[2*]Institut de Minéralogie, de Physique des Matériaux et de Cosmochimie, UMR 7590, Sorbonne Université, Museum National d'Histoire Naturelle, CNRS, Paris, 75005, France.
[3*]Institute of Geochemistry and Petrology, ETH Zürich, Clausiusstrasse 25, Zürich, 8092, Switzerland.
[4*]CRPG, CNRS, UMR 7358, 15 rue Notre Dame des Pauvres, Vandoeuvre-lès-Nancy, 54500, France.

*Corresponding author(s). E-mail(s): charnoz@ipgp.fr; Contributing authors: jerome.aleon@mnhn.fr ; chaussidon@ipgp.fr; psossi@ethz.ch; yves.marrocchi@univ-lorraine.fr; franco@ipgp.fr;

Keywords: Chondrites, condensation sequence, Solar Nebula

This file contains additional information concerning :

- The Equilibrium Condensation Sequence
- The details of the Kinecond code
- Several tests of the code
- Additional thermodynamical calculations related to the condensation timescales of the Solar Nebula
- Matrix mosaics displaying Kinetic Condensation Sequences in the case of fast, moderate and slow nebular reactions for varying pressures and condensation timescales.
- Details of the calculation of the fO_2 of the kinetic condensates for different cooling scenarios.
- Calculations of mineralogies resulting from the condensation of gases with non-solar compositions or in shocks.
- Additional comments about the preservation of precursors.

1 The classical Equilibrium Condensation Sequence (ECS)

The equilibrium condensation sequence, for a gas of solar composition and at pressure P=0.001 bar is displayed below, with calculation method described in [1] and [2].

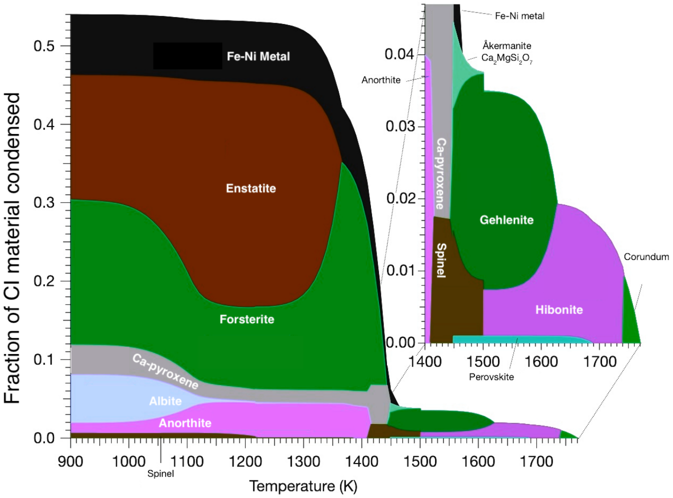

Supplementary Figure 1 Equilibrium condensation sequence for P=0.001 bar [1, 2].

2 Condensation of a gas with non-solar composition at equilibrium

In this work, to stay as close as possible to the "original" equilibrium condensation sequence, we have considered only the condensation of a gas with solar

composition. However, it is well known that the different chondrite classes have non-solar abundances of their elements and they can be significantly fractionated compared to the solar gas (see e.g. [3]). The origin of this fractionation is still very debated and could be linked to imperfect coupling of dust with gas during the evolution of the Solar Nebula [4, 5]. To test the effect of the condensation onto a gas of non-solar composition, we have considered condensation sequences with non-solar abundances. We start from a gas of solar composition, but we modified the abundances of Al, Mg, Fe, and S so that their ratio with respect to Silicon is the same as in selection of meteorites classes. Following the data compilation provided in [5] we have defined the following gases (see Table 1):

Gas Name	Al/Si	Mg/Si	Fe/Si	Reference
EH chondrite like	0.048	0.63	1.73	R1
H chondrite like	0.067	0.83	1.63	R1
L chondrite like	0.066	0.80	1.16	R1
LL chondrite like	0.063	0.81	0.98	R1
CO chondrite like	0.089	0.89	1.55	R2
CI chondrite like	0.078	0.89	1.74	R3

Supplementary Table 1 Composition of gases of non-solar composition. The abundances all atoms are solar except Al, Mg and Fe which abundances are determined so that their mass ratio with respect to silicon correspond to the values displayed in the above table. References R1: Wasson, J.T., Kallemeyn, G.W. 1988. Philosophical Transactions of the Royal Society of London Series A 325, 535. R2: Wolf, D., Palme, H. 2001. Meteoritics and Planetary Science 36, 559 and Palme (2001), R3: Palme, H. and O'Neill, H. St. C., 2014. Treatise on Geophysics Vol. 3. Elsevier pp. 1-39 and O'Neil (2014)

For all gases defined in the table above, we have tested different condensation sequences with fixed pressure (P=0.0001 bar).

We now turn to the case of the classical condensation sequence at equilibrium (ECS). We present below diverse condensation sequences at equilibrium, calculated with the FASTCHEM-COND CODE [6], for $P = 10^{-4}$ bar and for the gas compositions reported in SI Table 1.

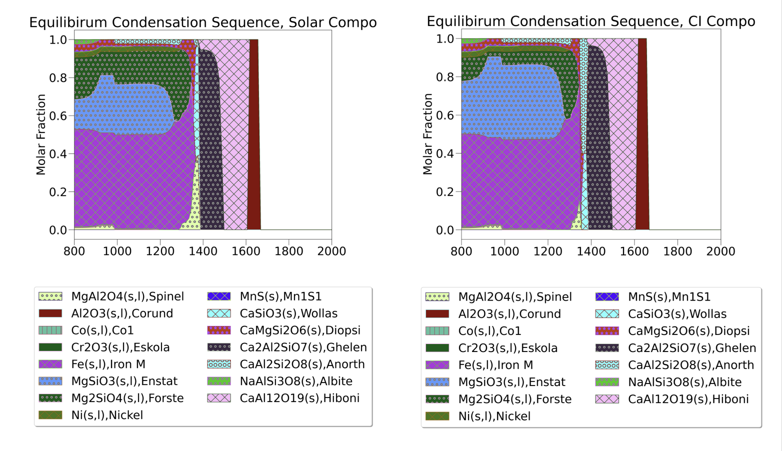

Supplementary Figure 2 Equilibrium Condensation Sequence (ECS) for a gas with Solar composition (left) and CI-chondrite like composition (right) (see SI Table 1 for the gas compositions). The horizontal axis stands for the temperature, and the vertical axis stands for the molar fraction of each mineral. The calculation was performed with the chemical equilibrium code FASTCHEM-COND [6]. The left graph should be compared to the kinetic condensation sequence of a gas of solar composition displayed in Figure 2 of the main article, and the right graph should be compared to the kinetic condensation sequence displayed in SI Figure 25.

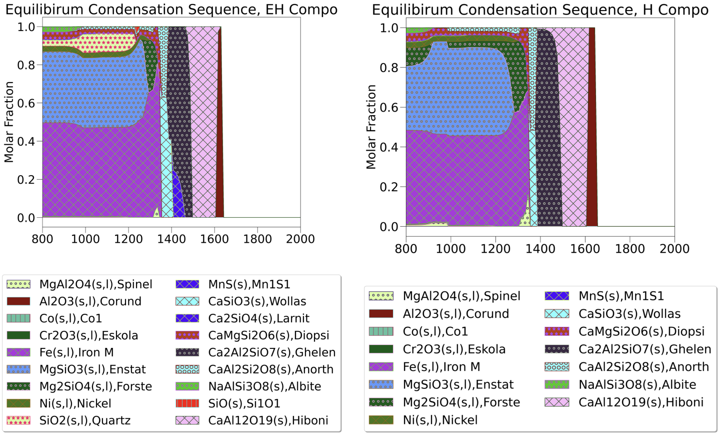

Supplementary Figure 3 Equilibrium Condensation Sequence (ECS) for a gas with EH-chondrite like composition (left) and H-chondrite like composition (right) (see SI Table 1 for the gas compositions). The horizontal axis stands for the temperature, and the vertical axis stands for the molar fraction of each mineral. The calculation was performed with the chemical equilibrium code FASTCHEM-COND [6]. The left graph should be compared to the kinetic condensation sequence of EH displayed in SI Figure 20, and the right graph should be compared to the kinetic condensation sequence displayed in SI Figure 21.

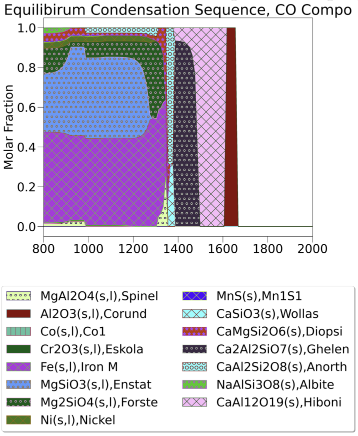

Supplementary Figure 4 Equilibrium Condensation Sequence (ECS) for a gas with CO-chondrite like composition (see SITable 1 for the gas compositions). The horizontal axis stands for the temperature, and the vertical axis stands for the molar fraction of each mineral. The calculation was performed with the chemical equilibrium code FASTCHEM-COND [6]. It should be compared to the kinetic condensation sequence displayed in SI Figure 24.

3 Detailed Method of calculation

3.1 `KineCond`: a Kinetic condensation model

`KineCond` is a code developed to calculate the time-dependent condensation processes of minerals in the Solar Nebula, which is dominated by Hydrogen. The elements considered in the system are H, He, O, Mg, Si, Fe, Al, Na, K, Ni, Ca, Cr, S, and C. Here, the term *element* designates an atomic species. The system consists of a gas in interaction with 39 minerals. Most of the condensation codes published in the past, and that allowed the investigation of the Equilibrium Condensation Sequence [1, 7–9] rely on the technics of Gibbs Energy Minimization (GEM). GEM calculates, for a given P and T, the most stable combination of minerals and gas. However, like any equilibrium calculation, it provides no information if the equilibrium state is established in a reasonable time, nor does it give the list of reactions through which the equilibrium state is realized (even though some additional physical arguments may help to determine those reactions, especially at high temperature when the number of minerals in presence is small). However, each reaction has its own kinetic, that depends on P, T, and the local abundances of all elements. Thus, in order to design a time-dependent (kinetic) code, we must adopt a different strategy, explicitly specify the list of all reactions considered, to advance all of them individually.

`KineCond` proceeds as follows: at each time step, we compute the number of atoms in the gas and the number of atoms in every mineral, keeping the total number of atoms constant. We assume that the pressure is constant and that only the temperature varies with time. The evolving variables of the system are: N_i^{gas} (the number of moles of each molecule i in the gas) and N_j^{min} (the number of moles of each mineral j in the system; see Table 2). We assume that gas-gas reactions are much faster than gas-mineral reactions and condensation reactions, so the gas molecular composition is always close to chemical equilibrium (then the gas molecular composition only depends on the current values of T and P as well as the number of moles of each element in the gas: N_i^{gas}). As we focus on gas-mineral processes, reactions are divided into two broad categories: condensation/evaporation reactions, and gas-mineral reactions. The rates of these reactions dictate the evolution of N_i^{gas} and N_j^{min}. The temperature varies linearly with time, dropping from 2000 K to 130 K on the time scale T_c (ranging from 0.01 to 2000 years). The system evolves as follows: At each time t we first calculate the molecular composition of the gas (excluding mineral condensation) by computing the chemical equilibrium of the gas at T (t) and P and with elemental abundances $N_i^{gas}(t)$. This is performed using the iconic `CEA-NASA` code (which stands for "Chemical Equilibrium with Application" distributed by NASA) [10], including about 1500 gas species. The instantaneous gas molecular composition is then used to compute the different condensation and gas-mineral reactions and the rate at which they proceed. We detail these calculations below.

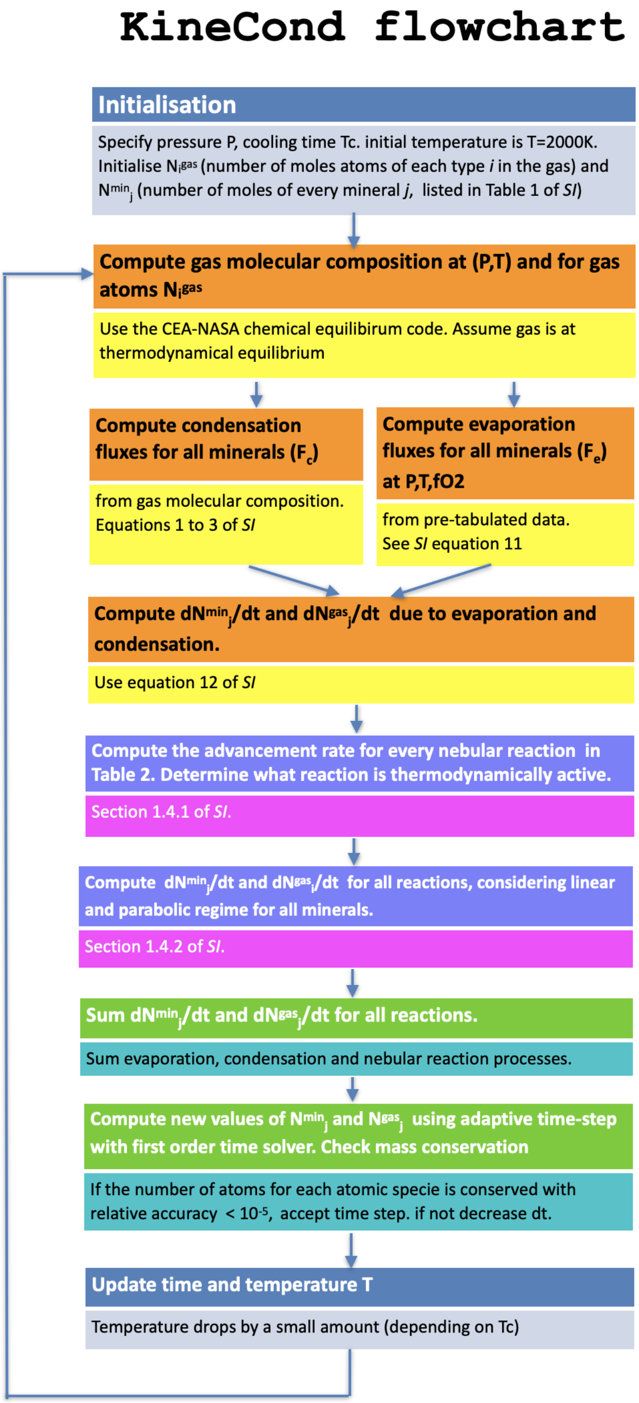
KineCond flowchart
Initialisation
Specify pressure P, cooling time Tc. initial temperature is T=2000K. Initialise N_i^{gas} (number of moles atoms of each type *i* in the gas) and N^{min}_j (number of moles of every mineral *j*, listed in Table 1 of *SI*)
Compute gas molecular composition at (P,T) and for gas atoms N_i^{gas}
Use the CEA-NASA chemical equilibirum code. Assume gas is at thermodynamical equilibrium
Compute condensation fluxes for all minerals (F_c)
from gas molecular composition. Equations 1 to 3 of *SI*
Compute evaporation fluxes for all minerals (F_e) at P,T,fO2
from pre-tabulated data. See *SI* equation 11
Compute dN^{min}_j/dt and dN^{gas}_j/dt due to evaporation and condensation.
Use equation 12 of *SI*
Compute the advancement rate for every nebular reaction in Table 2. Determine what reaction is thermodynamically active.
Section 1.4.1 of *SI*.
Compute dN^{min}_j/dt and dN^{gas}_j/dt for all reactions, considering linear and parabolic regime for all minerals.
Section 1.4.2 of *SI*.
Sum dN^{min}_j/dt and dN^{gas}_j/dt for all reactions.
Sum evaporation, condensation and nebular reaction processes.
Compute new values of N^{min}_j and N^{gas}_j using adaptive time-step with first order time solver. Check mass conservation
If the number of atoms for each atomic specie is conserved with relative accuracy $< 10^{-5}$, accept time step. if not decrease dt.
Update time and temperature T
Temperature drops by a small amount (depending on Tc)

Supplementary Figure 5 Flowchart of `KineCond` code. Orange/yellow boxes refer to condensation and evaporation processes, violet / pink refer to nebula reactions, green / blue refer to updates of mineral and gas abundances in the system and mass conservation checks, blue / gray are initial and final operations.

3.2 Condensation/evaporation reactions

We follow the condensation and evaporation theory presented in [11], [12] for forsterite in a H_2 gas, but we generalize it to many minerals. The net formation rate of a mineral j is the difference between an evaporation flux (J_j^e) and a condensation flux (J_j^c). Each of them must be computed explicitly. In the gas, the flux of any element E across a unit surface (in $moles/s/m^2$) is calculated using the kinetic theory of gases [11, 12]:

$$J^c(E) = \sum_m \frac{\nu_m^E P_m}{(2\pi\mu_m RT)^{1/2}} \tag{1}$$

where m is any gas molecule and ν_m^E is the stoichiometric coefficient of element E in molecule m, P_m is the partial pressure of molecule m ,μ_m the molar mass and R is the ideal gas constant. The partial pressures of gas molecules, P_m, are obtained by running the CEA-NASA code. We now consider a mineral j with formula $\left\{\alpha_j^E E\right\}$ with α_j^E the stoichiometric coefficient of element E in mineral j. The condensation flux of the mineral j (J_j^c) is determined by the smallest flux (over all elements E entering in its composition) so that :

$$J_j^c = \gamma_{E,j} min_E \left(\frac{J^c(E)}{\alpha_j^E}\right) \tag{2}$$

$\gamma_{E,j}$ is the sticking efficiency of atom E on mineral j. It ranges from 0 to 1 and is poorly constrained. Here we set $\gamma_{E,j} = \gamma = 0.1$, as a standard value, for all minerals and all atomic elements.

The evaporation flux of the mineral j (J_j^c) is given by [13]:

$$J_j^e = \frac{P_j^{sat}}{(2\pi\mu_j RT)^{1/2}} \tag{3}$$

where P_j^{sat} is the saturating vapor pressure of mineral j at temperature T. P_j^{sat} is easily defined in vacuum and for a mineral that has an equivalent gas form with same formula (like H_2O or Fe).

However, most minerals do not have a corresponding gas form (like Mg_2SiO_4), making Equation 3 not directly applicable to every mineral. In addition, the presence of the surrounding gas (mainly H and He) modifies the chemical equilibrium and must be taken into account when computing the saturation vapor pressure. So, Equation 3 is not directly applicable to compute the evaporation flux of all minerals immersed in a surrounding gas rich in H and He. To compute J_j^e from the kinetic theory of gases, we follow a strategy

developed in [11] and in [12] where the evaporating flux is explicitly computed without the need to compute the saturating vapor pressure. When a mineral is in equilibrium with its surrounding gas, the evaporating and condensation fluxes exactly balance (Equations 2 and 3).

So, determining the evaporation flux of the mineral j is equivalent to determining the condensation flux of the mineral j in equilibrium with the surrounding gas at pressure P and temperature T. Following [11], we provide a simple example to determine the evaporation flux of Mg_2SiO_4. We consider the following simplified reaction system:

$$Mg_2SiO_4(s) = 2Mg + SiO + 3/2O_2 \tag{4}$$

$$H_2 + 1/2O_2 = H_2O \tag{5}$$

Assuming that H_2 and He are the dominant species, $K_a(T)$ and $K_b(T)$ the equilibrium constants of reactions 4 and 5, and assuming that Mg, Si and O are in stoichiometric proportions, we have the following partial pressures relations :

$$P_{H_2} + P_{He} = P \tag{6}$$

$$P_{SiO} = \frac{Ka(t)}{P_{Mg}^2 P_{O_2}^{3/2}} \tag{7}$$

$$Kb(t) = \frac{P_{H2O}}{P_{H2} P_{O2}^{1/2}} \tag{8}$$

$$P_{SiO} = 1/2 P_{Mg} \tag{9}$$

$$P_{O_2} = 2/3 P_{SiO} \tag{10}$$

where in reaction 7 it is assumed that the condensate is ideal (activity coefficient=1) with molar fraction=1. We have 5 unknowns (the gas partial pressures) and 5 equations (6 to 10) so that all pressures can be determined. The evaporation rate of Mg_2Si0_4 is then determined by computing $J^c_{Mg_2SiO_4}$ from the different partial pressures (using Equation 2) and using the equilibrium relation $J^e_{Mg_2SiO_4} = J^c_{Mg_2SiO_4}$. This method can be generalized for any mineral immersed in H_2. In `KineCond` we have used a numerically intensive numerical approach and tabulated the evaporative flux of the 39 minerals at equilibrium with the surrounding H_2 gas using Equation 2. The pressure P of the gas H_2 varied from 10^{-10} bar <P< 0.1bar and the temperature T to $130K < T < 2200K$ and varying fO_2. The solution of the equilibrium chemical network was determined using the CEA-NASA code. All of these data are compiled into lookup tables and are interpolated at current (T, P, fO_2). So in `KineCond` the evaporative flux mineral j is obtained by reading a precalculated lookup table and interpolated between tabulated values at current values of ln(P), T and ln(fO_2). The result is multiplied by γ, so the sticking efficiency (assumed to be 0.1) is such that :

$$J^e_j = \gamma F_j(P, T, fO_2) \tag{11}$$

Supplementary Table 2 List of minerals and their thermodynamical data. References are : G91 [14], G96 [15], B83 [16], B85 [17], B88 [18],SUPCRT [19], C98 [20], P83 [21] P87 [22],

Formula	Name	Reference	Formula	Name	Reference
Al_2O_3	Corundum	G96	$FeAl_2O_4$	Hercynite	SUPCRT
$CaAl_{12}O_{19}$	Hibonite	B83	FeO	Wustite	C98
$CaSiO_3$	Wollastonite	SUPCRT	FeS	Troilite	C98
$Ca_2Al_2SiO_7$	Ghelenite	B88	$FeSO_4$	Ferrous Sulfate	C98
$Ca_2MgSi_2O_7$	Akermanite	B88	H_2O	Water Ice	G91
Ca_2SiO_4	Larnite	SUPCRT	KSi_3O_8	Microcline	SUPCRT
$Ca_3Al_2Si_3O_12$	Grossular	SUPCRT	K_2O	K oxide	G96
$Ca_3Fe_2Si_3O_12$	Andradite	SUPCRT	$K_2Si_2O_5$	K silicate	G82
$CaAl_2O_4$	Krotite	SUPCRT	Mg_2SiO_4	Forstertite	C98
$CaAl_2Si_2O_8$	Anorthite	SUPCRT	$Mg_3Si_2O_9H_4$	Lizardite	SUPCRT
$CaAl_4O_7$	Grossite	B85	$MgAl_2O_4$	Spinel	C98
$CaMgSi_2O_6$	Diopside	B88	$MgCr_2O_4$	Magnesiochromite	SUPCRT
CaS	Oldhamite	G96	$MgSiO_3$	Enstatite	C98
Cr_2O_3	Eskolaite	G82	$NaAlSiO_4$	Nepheline	SUPCRT
Fe	Iron Metal	C98	$NaAlSi_2O_6$	Jadeite	SUPCRT
Fe_2SiO_4	Fayalite	B88	Na_2O	Na oxide	G82
$Fe_3Al_2Si_3O_12$	Almandine	SUPCRT	$NaAlO_2$	NA Aluminate	C98
Fe_3O_4	Magnetite	P83	$NaAlSi_3O_8$	Albite	SUPCRT
$Fe_3Si_2O_9H_4$	Greenalite	SUPCRT	Ni	Nickel	G96
SiO_2	Quartz	G91			

The time derivative of number of moles of any mineral j due to competing condensation and evaporation processes is then :

$$\frac{dN_j^{min}}{dt} = S(J_j^c - J_j^e) \tag{12}$$

Where S is the surface of the grain gas in contact,J_j^c and J_j^e are given by equations 2 and 11, respectively. Our code implements a first-order time solver (Euler) and during a time step dt the number of moles of every mineral evolves according to Equation 12. To conserve the total number of moles of each element, the atoms released and removed from the gas (N_i^{gas}) are counted according to the stoichiometry of every mineral. We adopt an operator-splitting approach in which condensation reactions during the dt time step are treated first (Section 3.2), and gas-mineral reactions are treated in a second step (Section 3.4.2).

3.3 S: Surface of contact of grains with gas.

S depends on the grain radius (r) and the number of grains (N) so that $S \sim N4\pi r^2$. The self-consistent calculation of N and r necessitates the computation of the time-dependent nucleation process and requires the taking into account both the sticking and fragmentation processes of minerals during their settling and growth in the turbulent solar nebula. Currently, such a coupling (mineral condensation, coagulation, and fragmentation) has never been done, and some models take some processes into account (coagulation or fragmentation as in [23, 24], or metal condensation as in [25]). Models coupling dust settling, coagulation, and fragmentation show that dust size distribution reaches rapidly steady-state inward 10 Astronomical Unit (AU) in a few orbital periods [23, 26] and that what determines dust size is rather the equilibrium between coagulation and fragmentation in turbulence, rather than mineral growth. Following these lines, and to make the calculation tractable, we have simply used a characteristic dust size, which gives a gas-mineral surface of contact, rather than computing self-consistently a mineral growth model, and we limit ourselves to an order-of-magnitude calculation. In other terms, we assume that all minerals (despite their mass) have a surface in contact with the gas equivalent to a sphere with radius r = 10 μm (r is a free parameter of the model). This size is typical of the minerals observed in chondrites. The number of grains in the system, N, should be controlled by the number of nucleation sites that first appear in the gas. As aluminum is the most refractory atom in our model, we approximated $N \sim M_{Al}/(4/3\pi\rho_{Al}r^3)$ where M_{Al} is the total mass of Al in our system and ρ_{Al} is the density of aluminum, so S $=3M_{Al}/(\rho_{Al}r)$. This does not mean that all minerals are 10 micrometers radius, but rather that, on average, the total surface of contact of a mineral is equivalent to a population of minerals with an average of 10 μm radius (it could be fractal in shape). Of course, our calculation may not be accurate at the beginning of coagulation when the particle size is close to the monomer size, but [25] shows that changing the monomer size by a factor of 1000 only changes by 4% the growth time and does not change the final size. [27] finds that 70-micrometer-radius particles are formed in about 10 weeks (with a cooling rate about 100 kelvins/year at 10^{-4} bar, a pressure typical of the region of 0.1-1 AU), which is comparable to the orbital period at the distance of Mercury. Other works find the formation of 10 microns grains in a few orbital periods at 1 AU [23, 26] or at 5 AU [24]. Of course, larger minerals can be formed, but 10 microns is typical of what is observed in meteorites.

3.4 Gas-Mineral surface reactions (nebular reactions)

In addition to condensation and evaporation reactions, condensed minerals can also interact with the gas, leading to mineral transformation. We call "nebular reactions" those reactions by which a gas interacts with a pre-existing mineral (M1) and forms a new mineral (M2) and the expanse of M1. The number

of such reactions is potentially infinite, and for now `KineCond` implements reactions with the following generic form:

$$M1 + \beta_1 E1 + \beta_3 E2 \rightleftharpoons \alpha M2 \tag{13}$$

Where M1 and M2 represent two minerals, E1 and E2 represent any element in gas form, and α, β_1, β_2 are stoichiometric coefficients (normalized so that the stoichiometric coefficient of M1 is 1). Atomic elements E1 and E2 implied in the reaction, may participate in many molecular forms (Si could be in the molecular form SiO, Si, SiO_2 etc.). We show below that, when gas thermodynamic equilibrium is reached, then the generic form of reaction 13 is representative of all reactions with the form:

$$M1 + X1E1_{\beta 1} + X2E2_{\beta 2} \rightleftharpoons \alpha M2 + \beta 1X1 + \beta 2X2 \tag{14}$$

where X1 and X2 are arbitrary gas species. So, only detecting the equilibrium of reaction 13 allows us to detect simultaneously the equilibrium of all reactions in the form of reaction 14. This is illustrated in the following section with the example of one unique gas interacting with a mineral. This allows significant speed up of the calculation.

3.4.1 Reduced form of a reaction : simplification of the chemical system

Let's consider two minerals M2 and M1 which differ only by the content of one atom, let's say O, so that M2 contains more O than M1. Many reactions involving the mineral M1 and the gas can lead to the formation of M2 : for example, $Fe(s) + SO \rightleftharpoons FeO(s) + S$, $Fe(s) + OH \rightleftharpoons FeO(s) + H$, $Fe(s) + H_2O \rightleftharpoons FeO(s) + H2$, etc. However, when thermodynamic equilibrium is realized, all these reactions reach equilibrium under the same conditions, so they can all be replaced by a single simpler equation $Fe(s) + O \rightleftharpoons FeO(s)$. We call the latter the *reduced form* of all former reactions. The *reduced form* of a reaction, is the reaction where all spectators molecules has been removed. We show below that all reactions with the same reduced form have the same advancement rate, assuming the gas is at thermodynamical equilibrium.

This is demonstrated as follows: Let us consider a mineral M1, which interacts with a gas molecule of composition XO_β, with X representing any gas species and O representing any atom of interest (it could be oxygen, Si, etc.), and we have a mineral M2 with composition $M2 = M1 + \delta O$ with δ some stoichiometric coefficient. Let us assume that M1 interacts with the gas specie XO_β and produces mineral M2:

$$M1 + \alpha XO_\beta \rightleftharpoons \gamma M2 + \alpha X \tag{15}$$

γ is a stoichiometric coefficient to equilibrate the reaction. XO_β and X are gas species. For example the above reaction could represent :

$$Fe(s) + OH \rightleftharpoons FeO(s) + H \tag{16}$$

with X radical standing for H, and $\alpha = 1$,$\gamma = 1$, M1=Fe(S) and M2=FeO(s) (s=solid).

By definition for reaction 15 $\Delta G1 = \Delta^0 G1 + RT\ln(Q)$ but at equilibrium we have $\Delta G1 = 0$ and $Q = K1$ (K1 = reaction constant of reaction 15) so we get :

$$K1 = e^{-\Delta^0 G1/RT} \tag{17}$$

By the law of mass actions, at equilibrium :

$$\frac{P_X^\alpha * a(M2)^\gamma}{a(M1) * P_{XO_\beta}^\alpha} = e^{-\Delta^0 G1/RT} \tag{18}$$

where P_X and P_{XO_β} are the partial pressures of gases X and XO_β, and a(M1) and a(M2) are the activities of minerals M1 and M2. So to compute the equilibrium of the reaction 15 it seems we would need to know the partial pressures of X and the partial pressures of the gases XO_β. Thus, we would have to write as many reactions 15 as gas species X in the system (X=Si for SiO, X=C for CO_2, X=H2 for H_2O, X=H for HO etc...) .

But in fact we can do much simpler because all possible reactions, implying atom O, are linked when the gas is at thermodynamic equilibrium (as we always assume in `KineCond`) because the following homogeneous gas reaction is also at equilibrium :

$$XO_\beta \rightleftharpoons X + \beta O \tag{19}$$

If K2 is the reaction constant of Reaction 19 and $\Delta^0 G2$ is its free energy of formation then we have

$$\frac{P_X P_O^\beta}{P_{XO_\beta}} = e^{-\Delta^0 G2/RT} \tag{20}$$

From Equation 20 we can isolate P_{XO_β}

$$P_{XO_\beta} = P_X P_O^\beta e^{+\Delta^0 G2/RT} \tag{21}$$

and insert back into Equation 18 we get :

$$\frac{P_X^\alpha * a(M2)^\gamma}{P_X^\alpha P_O^{\alpha\beta} * a(M1)} e^{-\alpha\Delta^0 G2/RT} = e^{-\Delta^0 G1/RT} \tag{22}$$

Now, simplifying the above equation and assuming that a(M1)=(M2) = 1 (ideal solutions), we get :

$$\frac{1}{P_O^{\alpha\beta}} = e^{+\alpha\Delta^0 G2/RT - \Delta^0 G1/RT} \tag{23}$$

Taking the logarithm on both sides :

$$-\alpha\beta\ln(P_O) = \frac{-\Delta^0 G1 + \alpha\Delta^0 G2}{RT} \tag{24}$$

This transforms into

$$-\alpha\beta \ln(P_O) = \frac{(-\alpha G^0_X - \gamma G^0_{M2} + \alpha G^0_{XO_\beta} + G^0_{M1}) + \alpha(G^0_X + \beta G^0_O - G^0_{XO_\beta})}{RT} \tag{25}$$

which simplifies to

$$\alpha\beta \ln(P_O) = \frac{-G^0_{M1} + \gamma G^0_{M2}}{RT} \tag{26}$$

where we have used the unusual (but convenient) convention that the gas atomic forms are the reference forms, so that $G^0_O = 0$ [6, 28]. So we see from Equation 26 that the sole knowledge P_O is enough to determine if all reactions in the form of reaction 15 are at equilibrium. So, it is enough to replace all these reactions by a single one in the form :

$$M1 + \beta_1 O \rightleftharpoons \gamma M2 \tag{27}$$

with $\beta_1 = \alpha\beta$ is the total number of O atoms provided by the gas. Following Equation 26 we can introduce Q, the degree of advancement of the reaction so that :

$$\ln(Q) = \frac{\gamma G^0_{M2} - G^0_{M1}}{RT} - \beta_1 \ln(P_O) \tag{28}$$

When $\ln(Q) < 0$ then M2 is formed.

Above, the case of one single incoming gas was presented as an example. In the rest of this work, we consider two incoming gases, so that the equivalent reactions of many gas-mineral reactions implying two gases can be written in the generic following form:

$$M1 + \beta_1 A + \beta_2 B \rightleftharpoons \alpha M2 \tag{29}$$

With A and B representing atoms in the gas, and $\alpha, \beta 1, \beta 2$ representing stoichiometric coefficients. The corresponding Q is then written:

$$\ln(Q) = \frac{\alpha G^0_{M2} - G^0_{M1}}{RT} - \beta_1 \ln(P_A) - \beta_1 \ln(P_B) \tag{30}$$

with P_A and P_B representing the partial pressures of atoms A and B in their monatomic form in the gas.

For now, we are limited to reactions in which mineral M1 does not lose atoms to the gas. If M1 did, this could be an incongruent evaporation process, and this nebular reaction would be inconsistent with our hypothesis of treating condensation/evaporation as congruent processes using the Hertz-Knudsen formalism described above. Using combinatorial analysis, we found that 55 reactions of this type are possible with our mineral selections (Table 2). After many tests, only 38 reactions were kept, the others playing a more minor or no role (Table 3).

For a given T and for a given gas composition, we first compute if any reaction listed in table 3 is kinetically possible, that is, if the mineral M2 is formed (ln(Q)$\leq$ 0). To easily calculate the $\Delta^0 G$ of the formation of minerals M1 and M2 and the constant and the reaction constant K (T), we follow the approach of [28] and [6] where the reference elements are chosen to be in monoatomic gaseous form[28]. All G^0 are first corrected according to this convention. They are tabulated in the supplementary material of two studies [6, 29]. If ln(Q) $<$ 0 is detected, then the reaction favors the formation of mineral M2, and we can proceed to compute the rate at which it occurs.

3.4.2 Rate of nebular reactions

For computing the reaction rate, we follow an approach inspired by [30] called SCT (meaning: Simple Collisional Model), but modified to take into account published improvements in reaction rate on the surface of the minerals. We first determine the flux of the E1 and E2 incoming elements (using Equations 1) called $J^c(E1)$ and $J^c(E2)$. The c means *condensation*. We call them Elementary Fluxes. The smallest of the two fluxes (weighted by β_1 or β_2) controls the rate of progression of the reactions. So, if the reaction is thermodynamically possible, then the rate at which mineral M1 appears (and mineral M2 disappears) is:

$$\frac{dN_{M1}^{min}}{dt} = -S * min\left[J^c(E1)/\beta_1, J^c(E2)/\beta_2\right] \tag{31}$$

$$\frac{dN_{M2}^{min}}{dt} = -\alpha\frac{dN_{M1}^{min}}{dt} \tag{32}$$

Equation 31 assumes that all collisions lead the reaction and overestimates the reaction rate. So Equation 31 must be corrected. Laboratory experiments show that two regimes of reaction rates exist [31–34]: the linear regime and the parabolic regime. In the linear regime, each molecular collision has a certain probability of producing a chemical reaction, parameterized by the activation energy E_a^l. So the linear rate is :

$$\left.\frac{dN_{M1}^{min}}{dt}\right|_{linear} = -S * min\left[J^c(E1)/\beta_1, J^c(E2)/\beta_2\right] \times e^{-E_a^l/RT} \tag{33}$$

We recover here the SCT of [30]. However after a first period during which the reaction rim grows linearly with time at the mineral's surface , the reaction switches to a parabolic regime where atomic diffusion across the reactive rim limits the reaction rate, and the rim grows with the square root of time. In that case, the reactive layer with thickness H grows like $H^2 = k(T)t$ where k(T) is a diffusion coefficient that depends on temperature (m^2/s) and t is time [32]. It is usual to write $k(T) = Ce^{-E_a^p/RT}$ with C and E_a^p standing for a prefactor and an activation energy (in the parabolic regime). This process can

be described by a Fick diffusion law where the reaction rate (in $moles/m^2/s$) is

$$\frac{dN}{dt} = -Sk(T)\frac{dc(x)}{dx} \tag{34}$$

where c(x) is the concentration of mineral M2 at location x (x = 0 corresponds to the surface of mineral M1). In order to simplify the calculation we assume that c(x) drops linearly within M1, so that $dc/dx \sim 1/H$. So we solve :

$$\frac{dN}{dt} = -Sk(T)\frac{1}{H} \tag{35}$$

where H is the thickness of the mineral M2 layer above the M1 surface and S the grain surface. In `KineCond` H is calculated as $H = \mu_{M2}N_{M2}/(S\rho_{M2})$ where μ_{M2} and ρ_{M2} are the molar mass and density of mineral 2. To avoid nonphysical high reaction rates when H is close to 0 we bound the parabolic rate to be always smaller than the rate of incoming atoms to the M1 mineral's surface. So, the reaction rate in the parabolic regime reads:

$$\left.\frac{dN_{M1}^{min}}{dt}\right|_{parabolic} = -S \times min\left[\frac{k(T)}{H};\ min\left[J^c(E1)/\beta_1, J^c(E2)/\beta_2\right]e^{-E_a^l/RT}\right] \tag{36}$$

Unfortunately, there are only a handful of laboratory measurements [34] and most gas-mineral reactions are undocumented. For magnetite formation, laboratory experiments give $E_a^p \sim 90KJ/mol$ [31] while for Troilite (Fe_S) the activation energy in the parabolic rate is reported as $30KJ/mol < E_a^p < 70KJ/mol$ and in the linear regime is $28KJ/mol < E_a^l < 94KJ/mol$ [32]. These measurements were done in the (T,P) range of stability of both minerals. In contrast, the rate of forsterite to Enstatite (Mg_2SiO_4 + 1 Si + 2 O $\rightarrow$ $2MgSiO_3$) was measured at a temperature well above the stability of Enstatite or Forsterite ($>$ 1700K) and $E_a^p \sim 500KJ/mol$ was found [35]. For magnetite, troilite and enstatite activation energies, we use the laboratory values for the parabolic regime. For the linear regime and for all other reactions due to many uncertainties and lack of data [34] , we investigate different end-member scenarios defined below:

- Fast nebular reaction : (end member) $E_a^l = 0$ and $E_a^p = 0KJ/mol$
- Moderate nebular reaction : $E_a^l = 20Kj/ml$ and $E_a^p = 20KJ/mol$
- Slow nebular reactions : $E_a^l = 80Kj/mol$ and $E_a^p = 500KJ/mol$

The prefactor coefficient, C, is determined so that the linear and parabolic connect smoothly for a rim thickness of H=1 μm. This also prevents unrealistically high reaction rates for small values of H (a well-known problem of Fick's law). So C is equal to the linear rate divided by 1 μ m, that is, in the range of transition rim thicknesses measured for troilite formation [32]. For the enstatite formation experiment, the transition thickness is $<$ 10 microns [35].

Of course, the above procedure does not reflect the vast richness of gas-grain reactions and suffers from an important lack of experimental data. However, we have performed numerous tests, varying the values of E_a^l and E_a^p. We found that changing these values does not significantly impact our results as the processes investigated here are dominated by condensation processes, rather than gas-mineral surface interactions.

3.4.3 List of nebular reactions considered

By using combinatorial analysis we have determined all possible reactions (in their reduced form : meaning that spectator species are not written) implying 1 target mineral reacting with 2 gases. We found 55 possible reactions, listed below. In these 55 reactions, only 38 were found to be thermodynamically active in a system of condensing gas of solar composition. They are reported in Tab SI3.

- Mg2SiO4 + Si + 2O = 2MgSiO3 : Forstertite + Si + O = Enstatite
- 2Al2O3 + Ca + O = CaAl4O7 : Corundum + Ca + O = Grossite
- Al2O3 + Ca + O = CaAl2O4 : Corundum + Ca + O = Krotite
- Al2O3 + 2Na + O = 2NaAlO2 : Corundum + Na + O = Sod.Alum.
- Al2O3 + Mg + O = MgAl2O4 : Corundum + Mg + O = Spinel
- Al2O3 + Fe + O = FeAl2O4 : Corundum + Fe + O = Hercynite
- 6Al2O3 + Ca + O = Ca1Al12O19 : Corundum + Ca + O = Hibonite
- Fe + O = FeO : Iron Metal + O = Wustite
- Fe + Fe + 2O = 2FeO : Iron Metal + Iron Metal + O = Wustite
- Fe + 2Al + 4O = FeAl2O4 : Iron Metal + Al + O = Hercynite
- Fe + S = FeS : Iron Metal + S = Troilite
- Fe + Fe + 2S = 2FeS : Iron Metal + Iron Metal + S = Troilite
- Fe + S + 4O = FeSO4 : Iron Metal + S + O = Ferr.Sulfate
- 3Fe + 4O = Fe3O4 : Iron Metal + O = Magnetite
- 2Fe + Fe + 4O = Fe3O4 : Iron Metal + Iron Metal + O = Magnetite
- 2Fe + Si + 4O = Fe2Si1O4 : Iron Metal + Si + O = Fayalite
- CaAl4O7 + Ca + O = 2CaAl2O4 : Grossite + Ca + O = Krotite
- CaAl4O7 + 8Al + 12O = Ca1Al12O19 : Grossite + Al + O = Hibonite
- CaAl2O4 + 2Al + 3O = CaAl4O7 : Krotite + Al + O = Grossite
- CaAl2O4 + 2Si + 4O = CaAl2Si2O8 : Krotite + Si + O = Anorthite
- CaAl2O4 + 10Al + 15O = Ca1Al12O19 : Krotite + Al + O = Hibonite
- MgSiO3 + Mg + O = Mg2SiO4 : Enstatite + Mg + O = Forstertite
- NaAlO2 + 3Si + 6O = NaAlSi3O8 : Sod.Alum. + Si + O = Albite
- NaAlO2 + 2Si + 4O = Na1Al1Si2O6 : Sod.Alum. + Si + O = Jadeite
- NaAlO2 + Si + 2O = Na1Al1Si1O4 : Sod.Alum. + Si + O = Nepheline
- K2O + 2Si + 4O = K2Si2O5 : K2O1 + Si + O = K. silicate
- FeO + 2Al + 3O = FeAl2O4 : Wustite + Al + O = Hercynite
- FeO + S + 3O = FeSO4 : Wustite + S + O = Ferr.Sulfate
- 3FeO + O = Fe3O4 : Wustite + O = Magnetite
- 3FeO + 3Fe + 5O = 2Fe3O4 : Wustite + Fe + O = Magnetite

- 2FeO + Si + 2O = Fe2Si1O4 : Wustite + Si + O = Fayalite
- Na2O + 2Al + 3O = 2NaAlO2 : Na2O1 + Al + O = Sod.Alum.
- Cr2O3 + Mg + O = MgCr2O4 : Eskolaite + Mg + O = Magnesiochromite
- CaMgSi2O6 + Ca + O = Ca2MgSi2O7 : Diopside + Ca + O = Akermanite
- 2Ca1Si1O3 + Mg + O = Ca2MgSi2O7 : Wollastonite + Mg + O = Akermanite
- 3Ca1Si1O3 + 2Al + 3O = Ca3Al2Si3O12 : Wollastonite + Al + O = Grossular
- Ca1Si1O3 + Ca + O = Ca2Si1O4 : Wollastonite + Ca + O = Larnite
- SiO2 + 2Mg + 2O = Mg2SiO4 : Quartz + Mg + O = Forstertite
- SiO2 + Mg + O = MgSiO3 : Quartz + Mg + O = Enstatite
- 2SiO2 + 2K + O = K2Si2O5 : Quartz + K + O = K. silicate
- SiO2 + Ca + O = Ca1Si1O3 : Quartz + Ca + O = Wollastonite
- SiO2 + 2Fe + 2O = Fe2Si1O4 : Quartz + Fe + O = Fayalite
- SiO2 + 2Ca + 2O = Ca2Si1O4 : Quartz + Ca + O = Larnite
- FeS + 4O = FeSO4 : Troilite + O = Ferr.Sulfate
- Fe3O4 + Fe = 4FeO : Magnetite + Fe = Wustite
- Fe3O4 + 2Fe + O = 5FeO : Magnetite + Fe + O = Wustite
- Fe3O4 + 6Al + 8O = 3FeAl2O4 : Magnetite + Al + O = Hercynite
- Fe3O4 + 3S + 8O = 3FeSO4 : Magnetite + S + O = Ferr.Sulfate
- 2Fe3O4 + 3Si + 4O = 3Fe2Si1O4 : Magnetite + Si + O = Fayalite
- Ca1Al12O19 + 2Ca + 2O = 3CaAl4O7 : Hibonite + Ca + O = Grossite
- Ca1Al12O19 + 5Ca + 5O = 6CaAl2O4 : Hibonite + Ca + O = Krotite
- Na1Al1Si2O6 + Si + 2O = NaAlSi3O8 : Jadeite + Si + O = Albite
- Na1Al1Si1O4 + 2Si + 4O = NaAlSi3O8 : Nepheline + Si + O = Albite
- Na1Al1Si1O4 + Si + 2O = Na1Al1Si2O6 : Nepheline + Si + O = Jadeite
- Ca2Si1O4 + 2Al + 3O = Ca2Al2SiO7 : Larnite + Al + O = Ghelenite
- Ca2Si1O4 + Si + 2O = 2Ca1Si1O3 : Larnite + Si + O = Wollastonite

Supplementary Table 3 List of nebular reactions considered. Each reaction is a *reduced form* (i.e. spectator gas molecules are not written) and stands for all gas mineral-reactions, implying one mineral M1 reacting with one, or two, incoming gases to form one mineral M2. (s) means "solid form". See Section SI 3.4.1 and 3.4.3 for details.

1	$Fe(s) + S \rightarrow FeS(s)$
2	$Fe(s) + O \rightarrow FeO(s)$
3	$3\ FeO(s) + O \rightarrow Fe_3O_4(s)$
4	$2\ FeO(s) + Si + 2\ O \rightarrow Fe_2SiO_4(s)$
5	$Al_2O_3(s) + 2\ Na + O \rightarrow 2\ NaAlO_2(s)$
6	$NaAlO_2(s) + 3\ Si + 6\ O \rightarrow NaAlSi_3O_8(s)$
7	$NaAlO_2(s) + 2\ Si + 4\ O \rightarrow NaAlSi_2O_6(s)$
8	$Na_2O(s) + 2\ Al + 3\ O \rightarrow 2\ NaAlO_2(s)$
9	$CaAl_{12}O_{19}(s) + 2\ Ca + 2\ O \rightarrow 3\ CaAl_4O_7(s)$
10	$2\ Al_2O_3(s) + O + Ca \rightarrow CaAl_4O_7(s)$
11	$Al_2O3(s) + O + Ca \rightarrow CaAl_2O_4(s)$
12	$Al_2O3(s) + O + Mg \rightarrow MgAl_2O_4(s)$
13	$CaAl_2O_4(s) + 2\ Al + 3\ O \rightarrow 1\ CaAl_4O_7(s)$
14	$CaAl_4O_7(s) + 8\ Al + 12\ O \rightarrow CaAl_{12}O_{19}(s)$
15	$6\ Al_2O_3(s) + O + Ca \rightarrow CaAl_{12}O_{19}(s)$
16	$Mg_2SiO_4(s) + Si + 2\ O \rightarrow 2\ MgSiO_3(s)$
17	$MgSiO_3(s) + Mg + O \rightarrow Mg_2SiO_4(s)$
18	$SiO_2(s) + 2\ Mg + 2\ O \rightarrow Mg_2SiO_4(s)$
19	$SiO_2(s) + 1\ Mg + O \rightarrow MgSiO_3(s)$
20	$2\ SiO_2(s) + 2\ K + O \rightarrow K_2Si_2O_5(s)$
21	$SiO_2(s) + Ca + O \rightarrow CaSiO_3(s)$
22	$SiO_2(s) + 2\ Fe + 2\ O \rightarrow Fe_2SiO_4(s)$
23	$SiO_2(s) + 2\ Ca + 2\ O \rightarrow Ca_2SiO_4(s)$
24	$CaAl_4O_7(s) + Ca + O \rightarrow 2\ CaAl_2O_4(s)$
25	$CaAl_4O_7(s) + 8\ Al + 12\ O \rightarrow CaAl_{12}O_{19}(s)$
26	$CaAl_2O_4(s) + 2\ Al + 3\ O \rightarrow CaAl_4O_7(s)$
27	$CaAl_2O_4(s) + 2\ Si + 4\ O \rightarrow CaAl_2Si_2O_8(s)$
28	$CaAl_2O_4(s) + 10\ Al + 15\ O \rightarrow CaAl_12O_19(s)$
29	$Ca_2SiO_4(s) + 2\ Al + 3\ O \rightarrow Ca_2Al_2SiO_7(s)$
30	$Ca_2SiO_4(s) + Si + 2\ O \rightarrow 2\ CaSiO_3(s)$
31	$Mg_2SiO_4(s) + 2\ Ca + 3\ Si + 8\ O \rightarrow 2\ CaMgSi_2O_6(s)$
32	$Mg_2SiO_4(s) + 4\ Ca + 3\ Si + 10\ O \rightarrow 2\ Ca_2MgSi_2O_7(s)$
33	$Al_2O_3(s) + Mg + O \rightarrow MgAl_2O_4(s)$
34	$MgSiO_3(s) + Ca + Si + 3\ O \rightarrow CaMgSi_2O_6(s)$
35	$CaSiO_3(s) + Mg + Si + 3\ O \rightarrow CaMgSi_2O_6(s)$
36	$Ca_2MgSi_2O_7(s) + Mg + 2\ Si + 5\ O \rightarrow 2\ CaMgSi_2O_6(s)$
37	$NaAlO_2(s) + Si + 2\ O \rightarrow 1\ NaAlSiO_4(s)$
38	$3\ Fe(s) + 4\ O \rightarrow Fe_3O_4(s)$

3.5 Putting all things together

The system is initialized as follows :

- Specify pressure P and the number of moles of each element (=atoms). These parameters remain constant throughout the calculation.
- Specify the cooling time T_c in years. Temperature will decrease linearly from 2000K to 130K in time T_c. The equivalent cooling rate is thus $(2000-130)/T_c$ K/year.
- At time=0 and T=2000K it is assumed that all elements are in gas form and (number of gas moles are the N_i^g) and the number of moles of every mineral is 0 initially (N_j^m).

The code is then advanced from t = 0 to t $=T_c$ with time steps dt ($dt << T_c$). Each time step is decomposed as follows.

1. Compute the gas molecular composition at T and P (using only atoms present in gas form) using the CEA-NASA equilibrium code [10].
2. Compute the resulting gas atomic fluxes (Equation 1).
3. Condensation/Evaporation phase : Calculate the condensation and evaporation fluxes for every mineral (Equation 12, and evolve the number of minerals accordingly and the number of elements in the gas.
4. Nebular reactions phase : Determine what nebular reactions occur in the gas (Equation 28) among the list of reactions considered (Table 3). For all these reactions, compute the rate of production of mineral M2 and the rate of destruction of mineral M1 (use equations 33 or 36 for the linear or parabolic reaction rate model).
5. Update the number of elements in the gas, enforcing mass conservation.
6. Increment time and go back to the first step.

3.6 Validation Tests

We show here various tests against laboratory experiments (when available) that validate various aspects of the code.

3.6.1 Reproducing evaporation fluxes

In Figure 6 we compare the evaporation flux of forsterite in H_2 gas as computed in `KineCond` against laboratory experiments [12] and we do find good agreement at high and low temperature, and in H_2 rich or poor environment.

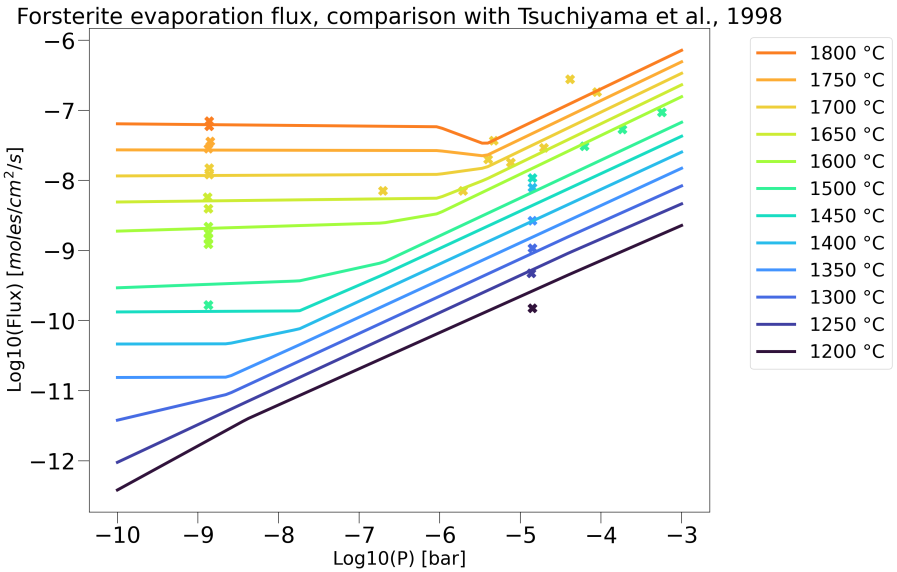

Supplementary Figure 6 Comparison of the Mg_2SiO_4 evaporation flux tabulated in `KineCond` (solid lines) and measured flux in laboratory experiments [12] (crosses). P is the total pressure of hydrogen species in the gas. At low hydrogen pressure, the vacuum evaporation flux is recovered, whereas at high hydrogen pressure, the effect of hydrogen increases the evaporative flux. Good match between the model and experimental data is observed.

3.6.2 surface reactions rates and reaction rim thickness

To test our procedure for nebular reactions (interactions of minerals with gas), we have numerically reproduced the conditions of the experiment presented in [35] that experimentally investigate the transformation of forsterite (Mg_2SiO_4) into enstatite ($MgSiO_3$). In this experiment, a forsterite crystal is immersed in a SiO_2 vapor produced by evaporation of a mineral of cristobalite. The pressure was controlled between 6×10^{-5} and 2×10^{-6} Torr and the temperature varies between 1468 ° C and 1514 ° C. At the surface of the forsterite mineral, a layer of forsterite is formed by reaction of Mg_2SiO4 with SiO in the gas. We have used our code to simulate this reaction. The surface S of the mineral was set to a cube of 1 mm in size. Only elements Mg, Si, O are considered in the system. The vapor pressure was fixed at 6×10^{-5} Torr and the vapor composition was set at 1 mole of Si for 2 moles of H. Condensation, evaporation and nebular reactions are fully coupled. The time evolution of the enstatite layer computed with `KineCond` is shown in Figure 7. For the activation energies, we have chosen the Slow Nebular Reactions (the closest to the activation energy reported in [35]). We find that in 10^5s to 10^6s a layer of enstatite , between 1 and 10 microns, forms on the surface of the forsterite crystal for temperatures of 1458 ° C and 1484 ° C with growth in the parabolic regime (slope ~ 0.5), which is very in good agreement with the experiment reported in [35] (Figure 4). The main difference is that we find that enstatite starts to evaporate at a temperature $>$

1500 °C, which limits the growth of the reaction rim to a fraction of a micron (green line in Figure 7). This behavior is probably due to our thermodynamic table, which predicts some evaporation of enstatite at these temperatures and pressures.

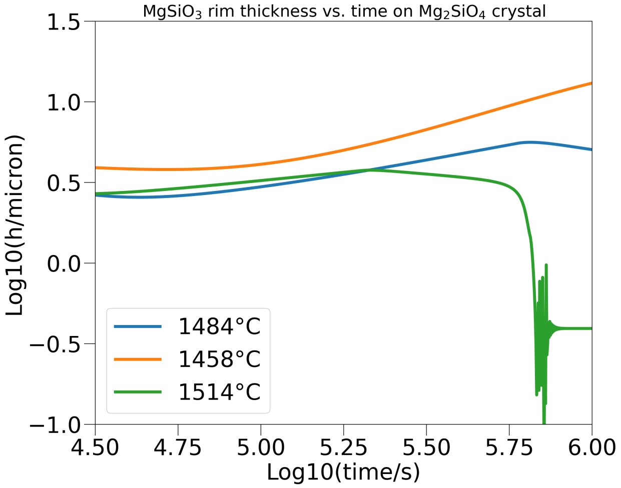

Supplementary Figure 7 Enstatite growth over a forsterite crystal in a SiO rich atmosphere, simulated with the `KineCond` code. Here, the active rim thickness is plotted as a function of time for different temperatures. Compare with Figure 4 of [35]

3.6.3 Reproducing the equilibrium condensation sequence

In our last test, we try to recover the Equilibrium Condensation Sequence (ECS). In principle, this is impossible because we do not compute the most stable mineralogical configuration (as it is done in the ECS), and all our minerals are competing simultaneously. However, on a very long timescale of evolution, for a very slowly cooling gas, we should recover a mineralogy that shares similarities with the condensation sequence, at least for the high temperature phases that evolve naturally faster. To accelerate the process of nebular reactions to converge toward chemical equilibrium at a fast pace we set all activation energies at 0, in both parabolic and linear regimes (this corresponds to our Fast Nebular Reactions case) and set the pressure at 0.001 bar and the condensation timescale at 1000 years (Figure 8). Comparison to the ECS at the same pressure shows good agreement : see, e.g., Figure 1 of [1] (SI Figure 1). The corundum condenses at $\sim$ 1740K, then replaced by grossite ($\sim$ 1670K). Then hibonite (1600K) and gehlenite (1530K) appear. The iron metal then begins to condense with nickel at 1450K, followed by forsterite (1430K) and enstatite (1335K). Below 1100K the mineralogy does not evolve anymore because evaporation timescales are extremely long. Finally, water ice appears at about 190K. The mineralogical sequence described here is very similar to the classic ECS at the same pressure. We recover similar condensation temperatures as in [1] and a mineralogical sequence for the different minerals at

least $1200K$. Below 1200K, however, we find differences because most minerals do no evaporate anymore, or at very low rate, or interact less efficiently with the gas. In contrast to ECS, we do find that typical CAI minerals survive at low temperature ($<$ 900K) in the form of diopside and spinel, representing approximately 10% of the bulk mass. Also Albite ($NaAlSi_3O_8$) does not form (it appears $\sim$ 1250K in ECS) because Al and Si are no longer available in the gas to react with sodium to form Albite. Between 200 and 1000K we recover the main mineralogical components of typical chondrites : iron metal, silicates (enstatite, olivine) and some high-temperature minerals.

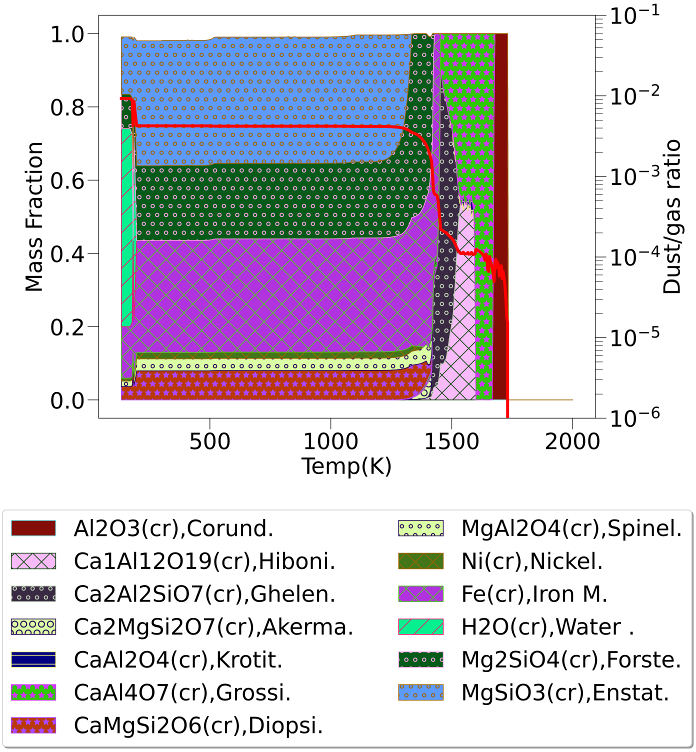

Supplementary Figure 8 Kinetic Condensation sequence computed with `KineCond` with fast nebular interactions, P=0.001 bar and condensation time=1000 year. Should be compared with classical condensation sequence in SI Figure 1. Minerals representing $>$ 1% of the total rock mass are displayed. The red line represent the dust to gas ratio and must be red on the right vertical axis

3.7 Limitations of `KineCond` and possible improvements

To make the calculation tractable, simplifications and approximations have been done and in `KineCond`, which are common to most condensation codes such as CEA-NASA [10], GGCHEM [29], FASTCHEM-COND [6] or ArCCoS [36]. We list them below:

1. **No taking into consideration of amorphous phases, whereas meteorite data show condensed amorphous phases [37]. However, there are only very few thermodynamic data on amorphous phases, and as for now this is a strong limitation for all condensation codes**.
2. No treatment of solid solutions: all minerals are considered as pure, so their activity is =1.
3. No treatment of alloys.
4. Simplistic treatment of nucleation (see section SI 3.3).
5. No treatment of gas dissolution into liquids.
6. The gas is assumed to be at thermodynamics equilibrium: it is the gas-mineral interactions that are out-of equilibrium and are time evolved.
7. The system is closed.

Our priority is the coupling of `KineCond` to a 1D disk model to allow us to treat open systems and explore elemental fractionation processes. We plan to include a nucleation algorithm, coupled to coagulation and fragmentation in turbulence in a second step. In a next step, a CMAS liquid model will be implemented.

4 Equilibration timescale in a minimum mass solar nebula

We can estimate the equilibration timescale, t of a mineral with radius r and density ρ considering that, to fully equilibrate a mineral with the surrounding gas, the mineral must be able to exchange its own mass with the surrounding gas. Calling F the flux of evaporation at temperature T and pressure P (in $moles/m^2/s$), we get $t \sim 4r\rho/(3\mu F)$ with μ as the molar mass. We show in SI Figure 9 some values of F extracted from our code from four minerals of importance (see Section SI.3.2)

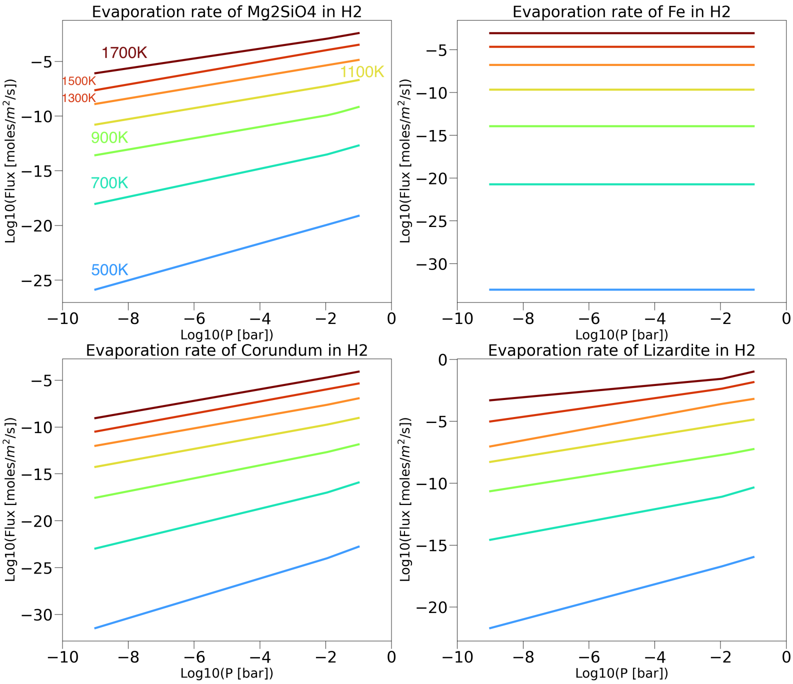

Supplementary Figure 9 Evaporation fluxes (in moles/m^2/s) computed in `KineCond` of a selection of minerals, assuming evaporation efficiency $\gamma = 0.1$, as a function of the H_2 ambient pressure. Top-left : forsterite, top right : iron metal, bottom-left : corundum, bottom-right : lizardite

We now consider a minimum mass solar nebula, with surface density $\Sigma(R) = 10^4$ Kg/m$^2(R/1AU)^{-3/2}$, temperature T(R)=800K$(R/1AU)^{-1/2}$. SI Figure 10 shows the pressure in the disk and SI Figure 11 the corresponding local equilibration timescales for Fe and forsterite grains of 10 micrometers radius.

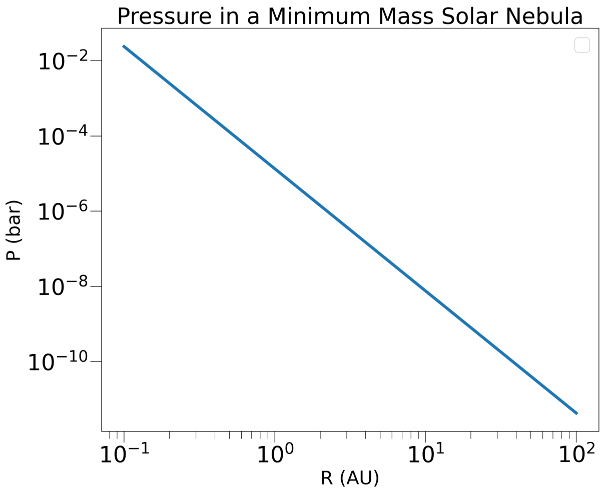

Supplementary Figure 10 Pressure versus distance in a Minimum Mass Solar Nebula, assuming T=800K at 1 AU.

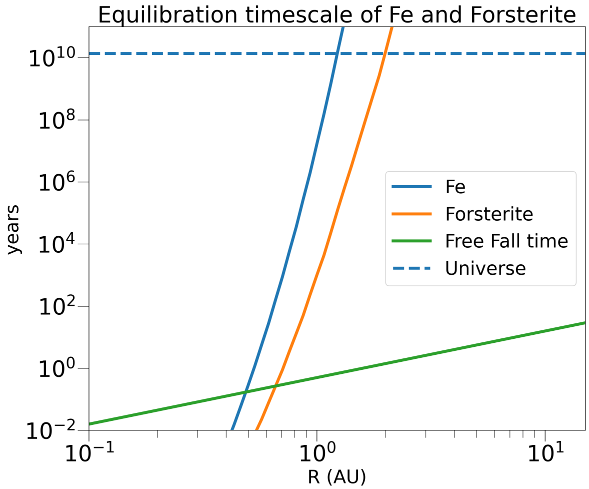

Supplementary Figure 11 Equilibration timescale of a 10 micron grain of metallic iron (blue) and forsterite (orange) as a function of distance in a minimum mass solar nebula. For comparison, the local free-fall timescale and age of the universe are displayed as well.

5 Fast versus slow condensation

We plot in (Fig. 12) the number of atoms in the gas for two different condensation histories: solid line for slow condensation with T_c=1000 years and dashed line for fast condensation with $T_c = 0.1$ year and $P = 10^{-4}$ bar. We see that for the slow cooling case, the main atoms leave the gas at high temperature, while for the fast cooling case most minerals are still mostly available in the

gas (> 0.5 in mole fraction) at temperatures significantly lower than 1000K, allowing for the condensation of low-temperature oxidized minerals such as magnetite, fayalite, or phyllosilicates. Jumps in the aluminum content of gas at $\sim$ 1650K are due to the transformation of corundum to grossite via nebular reactions followed by re-evaporation of a fraction of the grossite. This is an out-of-equilibrium effect of the competition of nebular reactions with condensation/evaporation processes.

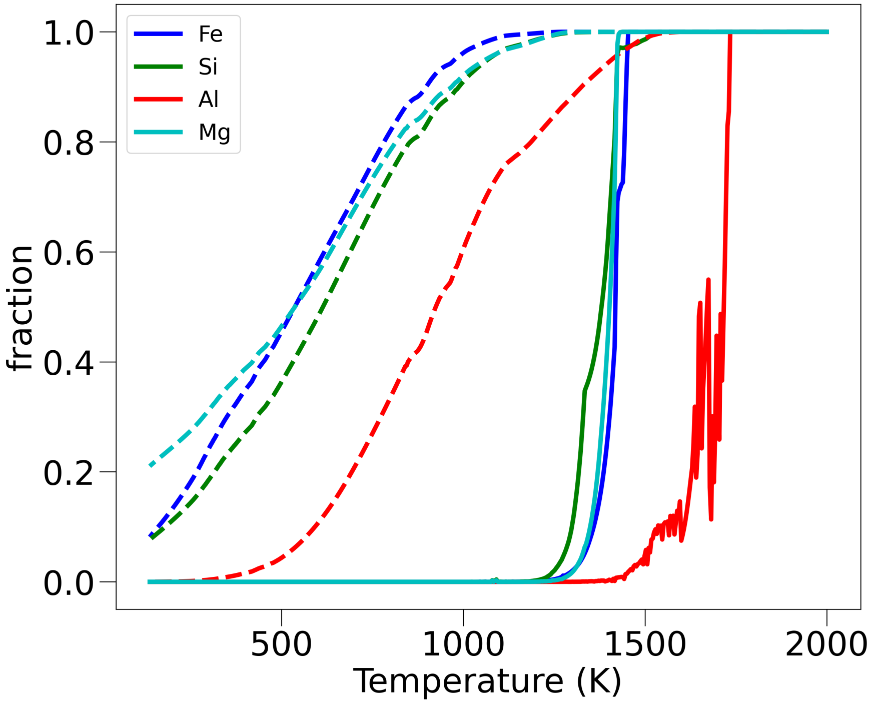

Supplementary Figure 12 Fraction of atoms remaining in the gas for P=10^{-4} bar for two condensation scenarios : solid line : Tc=1000 years, dashed line : Tc=0.1 year. The fast nebular case is shown.

6 Mosaics of condensation sequences: exploring -11 $\leq$X $\leq$+1

In this section we display, in the form of mosaics, our systematic exploration of the effect of cooling time (T_c) and pressure (P) on kinetic condensation sequences. Defining $X = \log_{10}(T_c/year) + \log_{10}(P/bar)$, we explore here the range -11 $\leq$X $\leq$+1. Each mosaic corresponds to a different kinetic rate of nebular reactions: fast nebular reactions (FNR), moderate nebular reactions (MNR), and slow nebular reactions SNR). These rates are defined in section SI 3.4. Inside each mosaic, each cell shows a condensation sequence (X-axis is temperature, Y-axis is mineral mass fraction, and the red line is the dust-to-gas ratio, to be red on the right scale). The meaning of each color is shown in SI Figure 16. Each mosaic is divided into 3 regions with different mineralogical properties: Type A (upper right triangle, with green contour), Type B (diagonal, with yellow contour), and Type C (lower left triangle, with blue contour). A high-resolution version of each mosaic, suited to be printed in large A0 format is available on this link .

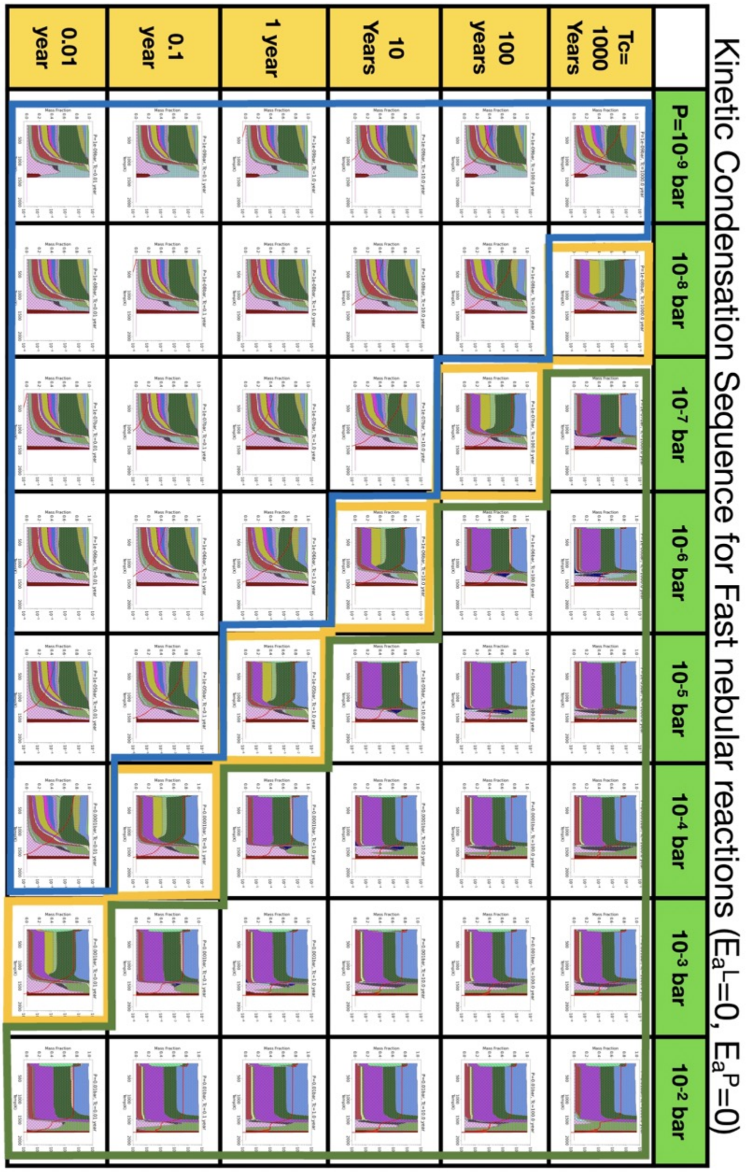

Supplementary Figure 13 Condensation sequences computed assuming fast nebular reactions processes. Condensation processes are not affected. The green, orange and blue contours delimit the regions of Type A, B and C mineralogies respectively. See figure 16 for color legend. A high resolution version, suitable to print on A0 poster format is available here link

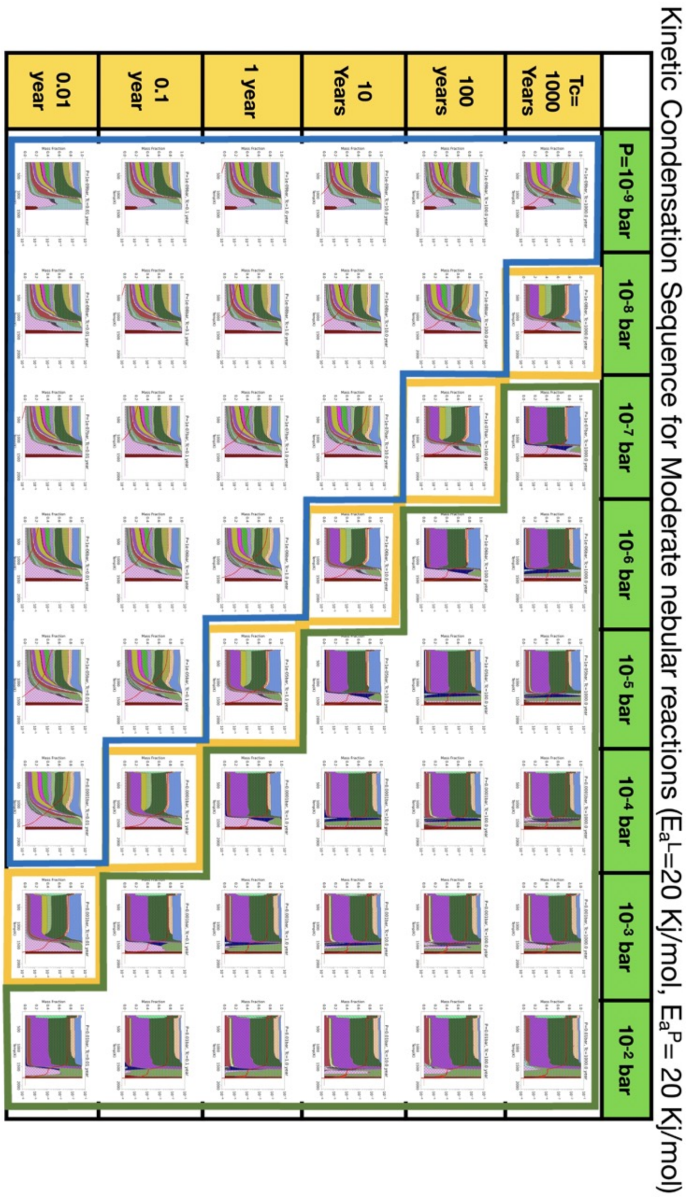

Supplementary Figure 14 Condensation sequences computed assuming moderate rate nebular reactions processes. Condensation processes are not affected. The green, orange and blue contours delimit the regions of Type A, B and C mineralogies respectively. See figure 16 for color legend. A high resolution version, suitable to print on A0 poster format is available here: link

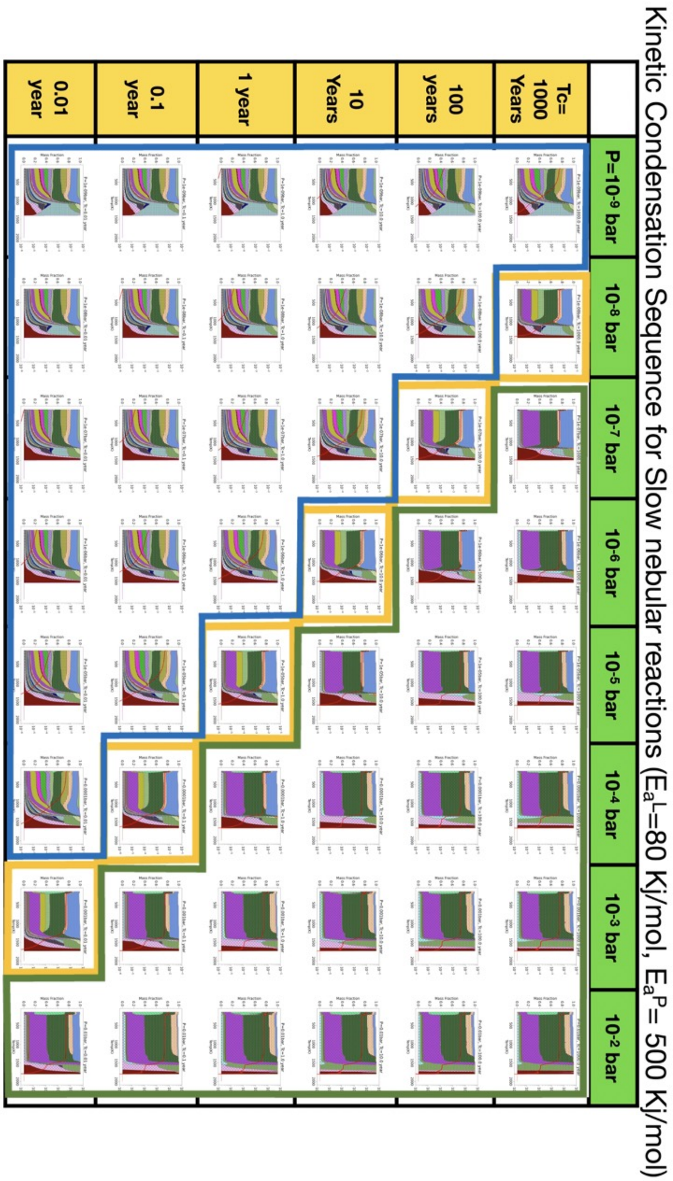

Supplementary Figure 15 Condensation sequences computed assuming slow rate nebular reactions processes. Condensation processes are not affected. The green, orange and blue contours delimit the regions of Type A, B and C mineralogies respectively. See figure 16 for color legend. A high resolution version, suitable to print on A0 poster format is available here link

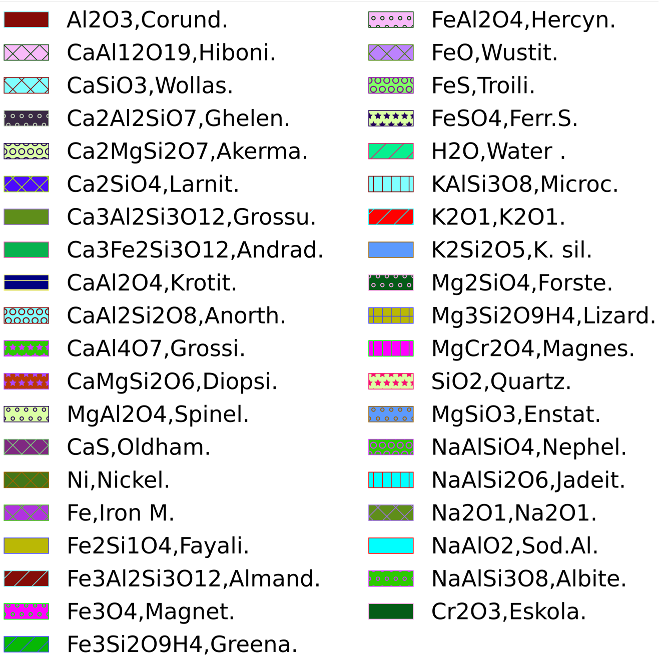

Supplementary Figure 16 Color legends for all minerals discussed in the present paper

7 Super Saturation in a cooling gas

The coefficient of super saturation, S, of a mineral with formula $A_\alpha B_\beta C_\gamma$ (with A,B,C standing for atoms and α, β, γ standing for stoichiometric coefficients) is computed as follows (see [28] their section 2.2 for a detailed discussion):

$$S = P_A^\alpha P_B^\beta P_C^\gamma e^{\frac{-\Delta G^0}{RT}} \tag{37}$$

with P_A, P_B, P_C standing for the partial pressures (in bars) of elements A,B,C in the gas and ΔG^0 is the Gibbs free energy of formation of the condensate. Here we use the convention [6, 28] that the reference form of any atom is the gas mono-atomic form. A mineral starts to condense when S $\geq$ 1. S is displayed for a selection of minerals, in a gas of solar composition (at chemical equilibrium among the gas species only) in SI Figure 17.

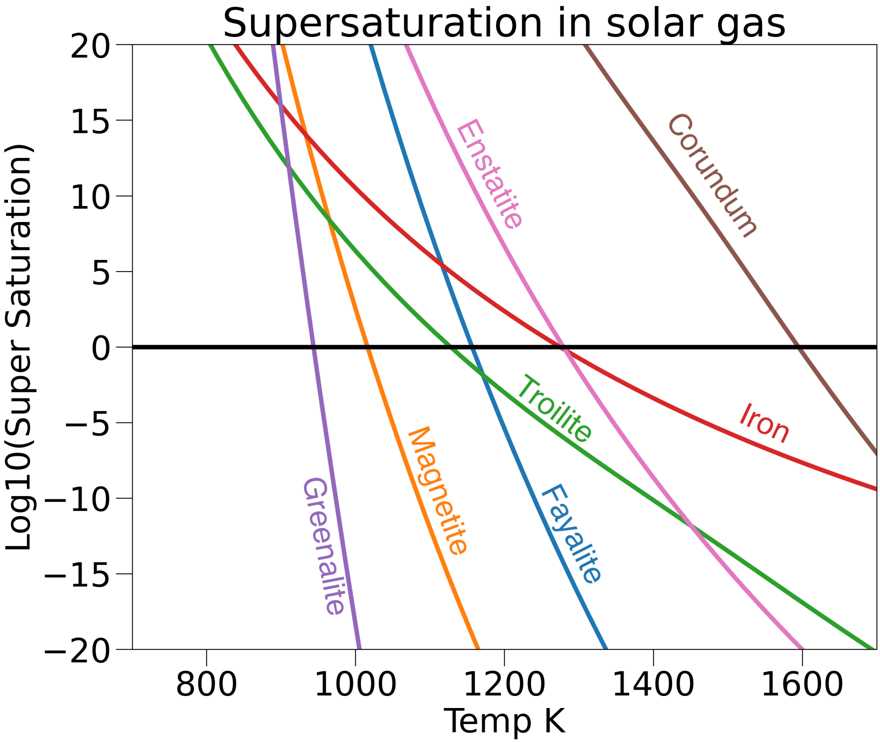

Supplementary Figure 17 Super saturation coefficient (S) for a selection of minerals in a gas of solar composition with pressure $P=10^{-5}$ bar as a function of temperature. Condensation occurs for $S>1$. The gas molecular composition, ignoring condensate formation was calculated with the CEA code [10].

8 Equivalent fO_2 of mineralogical types A, B and C

The equivalent fO_2 of the different calculated mineralogies was estimated as follows: for each mineral composition, we calculate the equilibrium mineralogy with the `factsage` software at a temperature of 1500K, a temperature at which a liquid-vapor equilibrium is established. The equivalent fO_2 is the partial pressure of O_2 in the vapor.

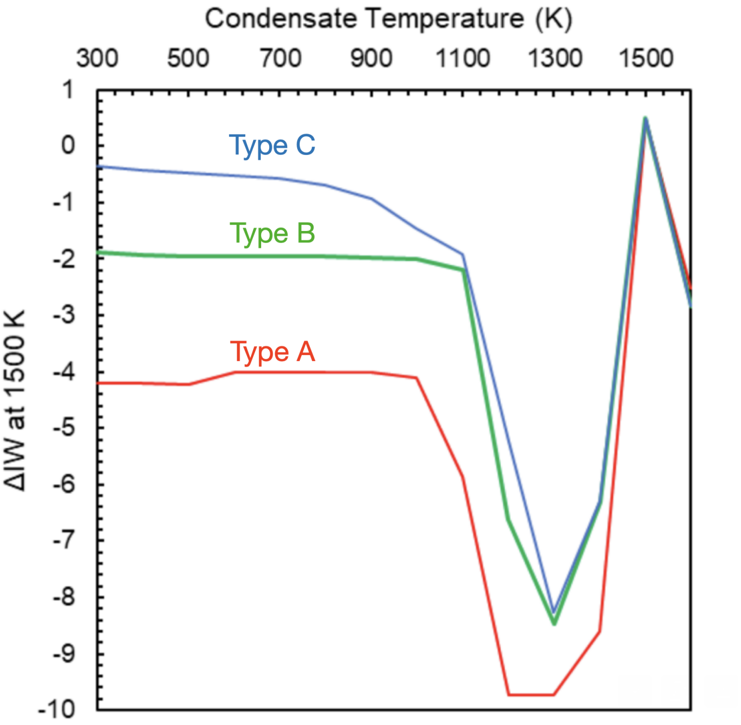

Supplementary Figure 18 Equivalent fo2 of re-equilibrated mineralogies for the three types A,B and C. The X axis stands for the temperature at which condensates are considered, while the Y axis gives the equivalent fo2 (with respect to the iron-wustite buffer). Here, the types A,B,C are computed using the fast nebular reaction case.

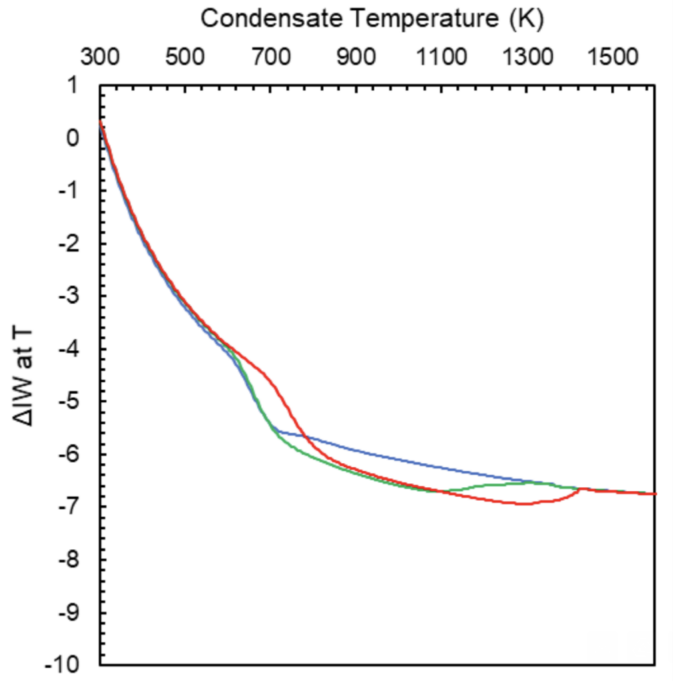

Supplementary Figure 19 Real fo2 of gas during the condensation sequence of types A,B,C objects.

9 Kinetic Condensation Sequence for non-solar gases

We investigate here the condensation of a gas of non-Solar Composition in the case of Kinetic Condensation Sequence (KCS). For all gases defined in SI Table 1 we present below the kinetic condensation sequence from 2000K to 150K and for two condensation times: 1 year or 0.1 and 0.01 year depending on cases. Again, we see that the resulting mineralogy changes only slightly between the different compositions. In the case of Kinetic condensation sequence a major effect of the condensation time is observed: compared left and right graph. Slow condensation is very similar to Equilibrium Condensation Sequence (compare figures below with figures in section SI 2), whereas fast condensation completely changes the mineralogy.

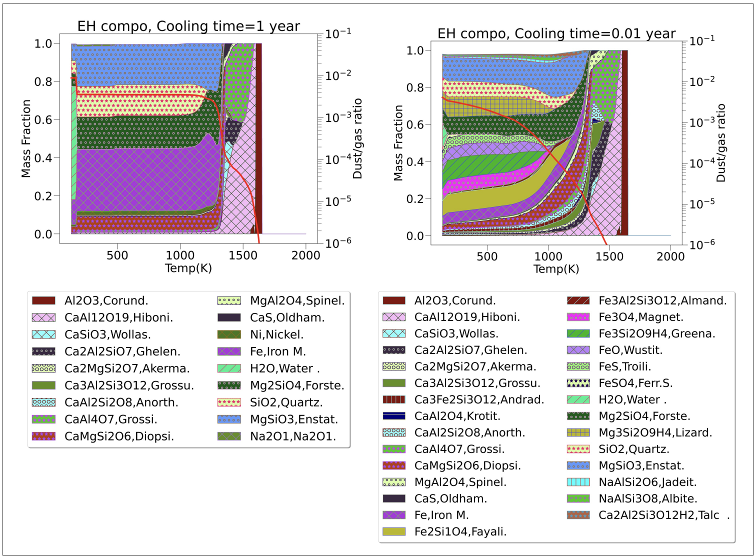

Supplementary Figure 20 Condensation sequence for a gas with enstatite like composition (see Table 1. Left: slow cooling in 1 year, right : fast cooling in 0.01 year, P=0.0001 bar

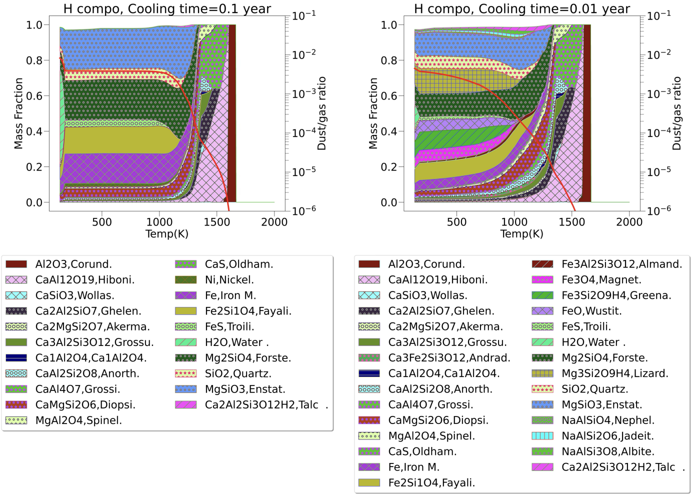

Supplementary Figure 21 Condensation sequence for a gas with Ordinary Chondrite H like composition (see Table 1. Left: moderate cooling in 0.1 year (corresponding to Type B mineralogy), right : fast cooling in 0.01 year, P=0.0001 bar

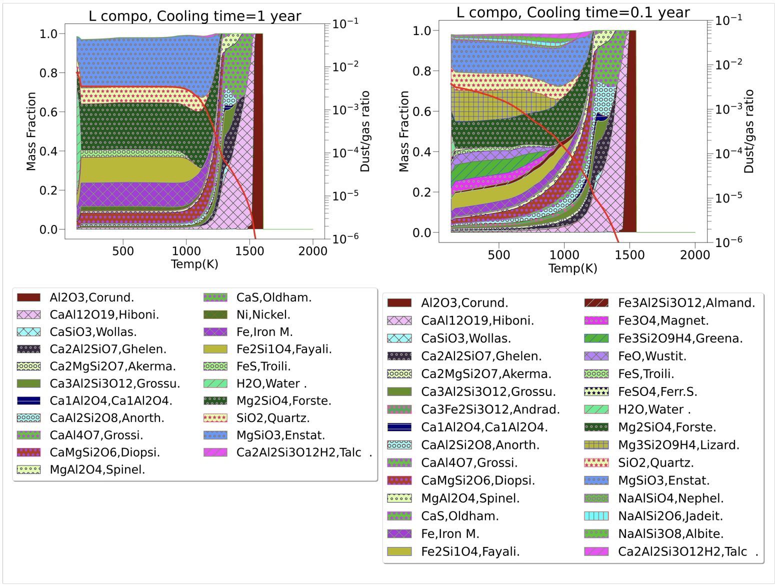

Supplementary Figure 22 Condensation sequence for a gas with L ordinary chondrite like composition (see Table 1. Left: cooling in 1 year corrresponding with Type B mineralogy, right : fast cooling in 0.1 year, P=0.0001 bar

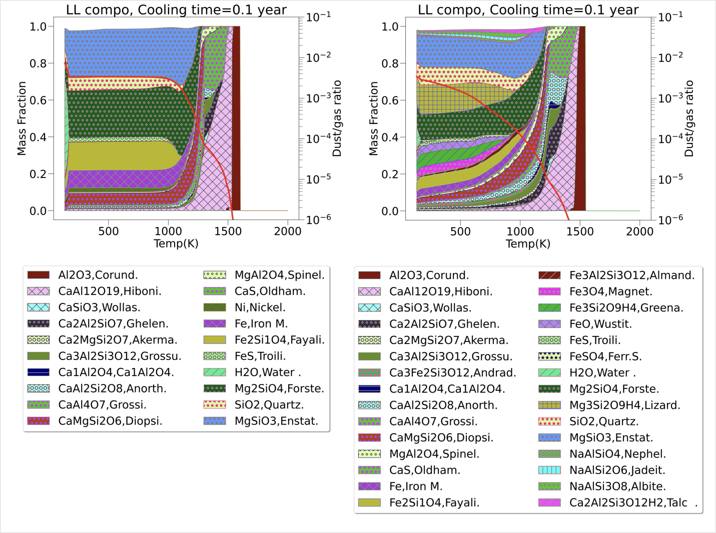

Supplementary Figure 23 Condensation sequence for a gas with LL ordinary chondrite like composition (see Table 1. Left: cooling in 0.1 year corresponding with Type B mineralogy, right : fast cooling in 0.1 year at P=0.0001 bar

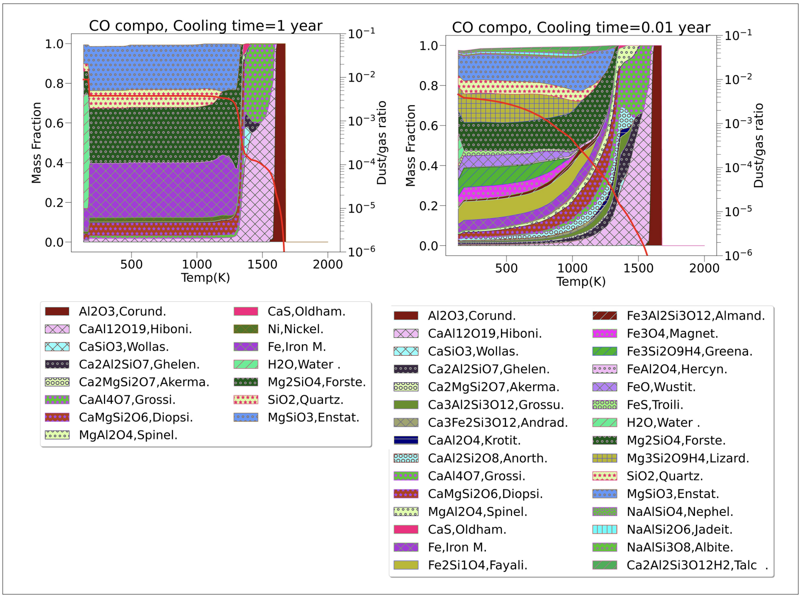

Supplementary Figure 24 Condensation sequence for a gas with Carbonaceous Chondrite CO like composition (see Table 1. Left: slow cooling in 1 year, right : fast cooling in 0.01 year, P=0.0001 bar

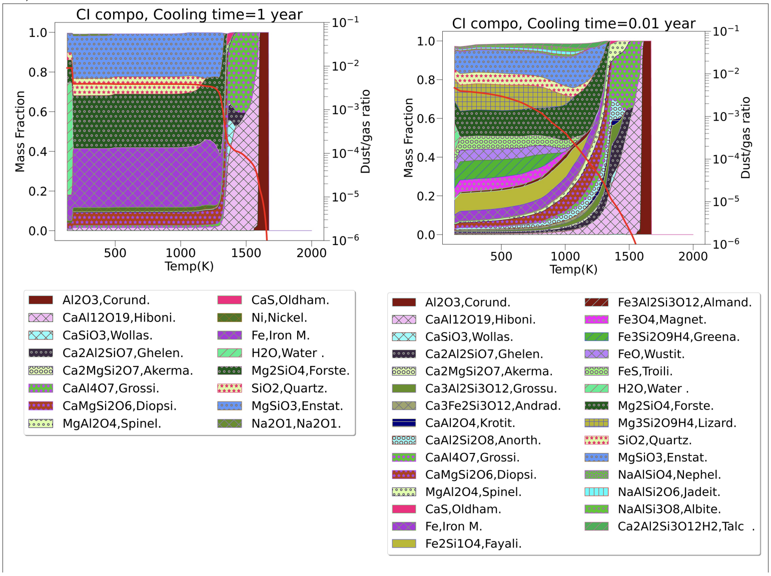

Supplementary Figure 25 Condensation sequence for a gas with CI carbonaceous chondrite like composition (see Table 1. Left: slow cooling in 1 year, right : fast cooling in 0.01 year, P=0.0001 bar

10 Thermal alteration in shocks

To show the effect of thermal alteration, melting, and evaporation, we simulated the impact of a thermal pulse on an already-formed mineral assemblage. Such thermal events are invoked, for example, to explain the formation of chondrules as solids traverse bow shocks around planetesimals in the protoplanetary disk [38, 39]. We performed three simulations under identical pressure conditions ($P = 10^{-4}$ bar) but with different thermal pulse intensities.

Each simulation begins with a phase of slow gas cooling over 1000 years (Figure SI.26, right column), leading to the condensation of a Type A (non-oxidized) mineralogy. The oxidation state just before the thermal pulse, at $t = 1000$ years, is indicated by a cross marker in the left column of Figure SI.26. At 1000 years a thermal pulse is applied, consisting of a brief heating phase lasting 0.01 year followed by a cooling phase lasting 0.1 year (visible in the right column of the figure). After the pulse, the system continues to cool down to 500 K over an additional 1000 years. In the three thermal histories considered here, the minimum and maximum temperature of the thermal pulse differ, resulting in different melting and cooling rate.

Depending on the maximum and minimum temperatures reached during the thermal pulse, the final oxidation state differs. This final state is marked with a square marker in the left column of Figure SI.26. In all cases, the final mineralogy corresponds to one of the three previously identified condensation types (A, B, or C).

In the first thermal history (panels a, b), the thermal pulse is weak and does not alter the mineralogy; the final state remains Type A, and the cross and square markers overlap. In the second case (panels c, d), the pulse modifies the mineralogy, resulting in a Type B composition, located near ordinary chondrites in the oxidation diagram. In the third case (panels e, f), the thermal pulse significantly oxidizes the condensates, shifting the final assemblage to a Type C mineralogy, close to the CO and CV chondrites.

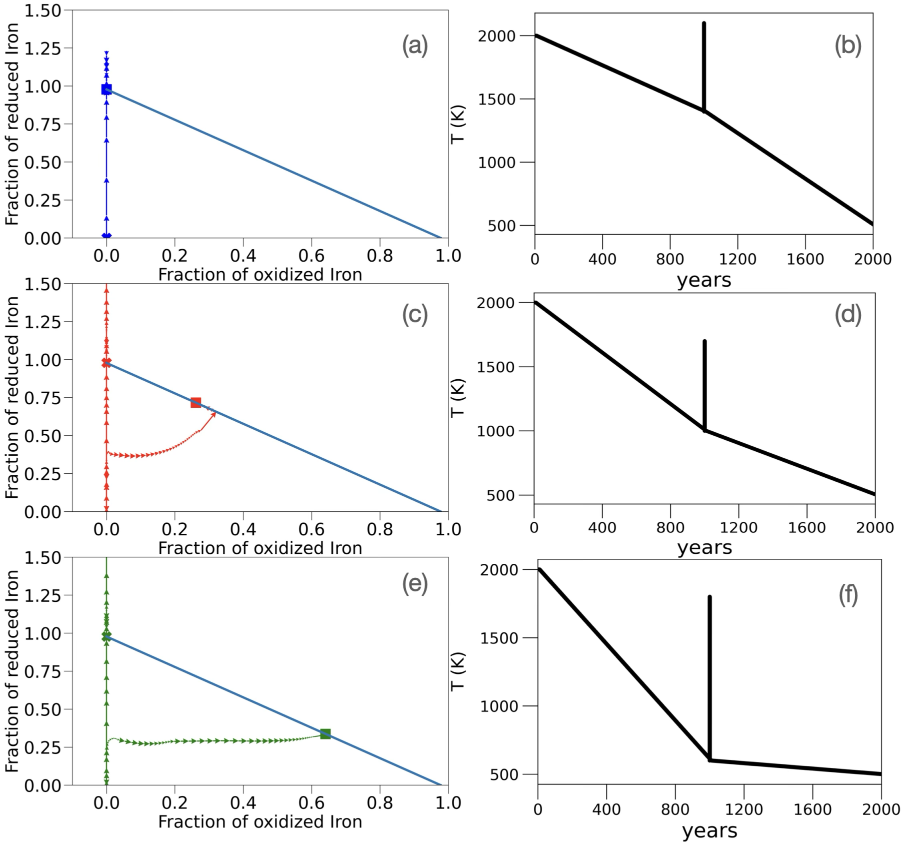

Supplementary Figure 26 3 different thermal histories of precursors (right column) and the corresponding evolution of the iron oxidation state of the condensates plotted in a Urey-Craig diagram (left column). (a,b) small thermal pulse that occurs when the precursors are still at high temperature, (c,d) moderate thermal pulse that occurs when the precursors are still at warm temperature, and (e,f) intense thermal pulse that occurs when the precursors are low temperature. The colored crossed marker shows the oxidation state of the system just before the thermal pulse occurs (at 1000 years), and the colored squared marker shows the oxidation state of the system at the end of the process. The solid blue line in the left column displays the solar Fe/Si ratio.

11 Sketch about the preservation of precursors

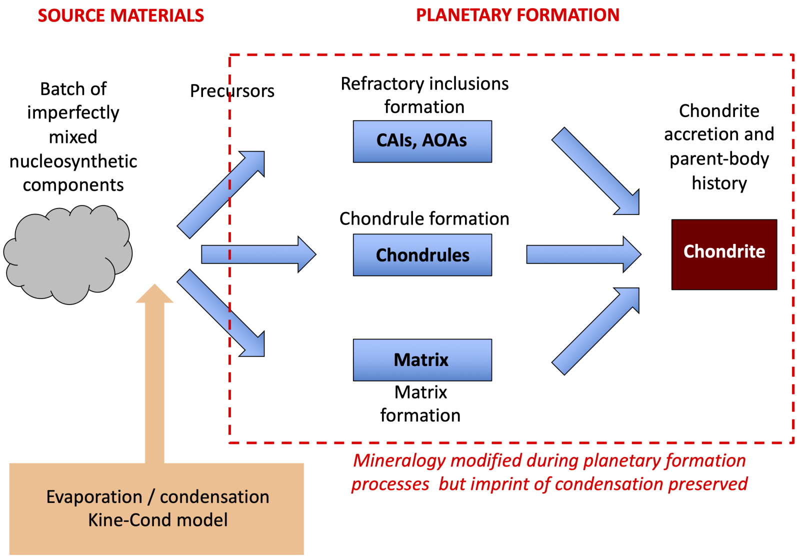

Supplementary Figure 27 Schematic description of a scenario in which kinetic condensation could have influenced the bulk chemistry of chondritic bodies from the early solar system. While the final mineralogy of chondrites is due to the accretion and subsequent parent-body modification of individual components (refractory inclusions, chondrules and matrix), each having their own history, the conditions of condensation of their precursors controlled their diversity and redox state, thus influencing the outcome of subsequent processes. For instance the proportion of reduced and oxidized precursors may have influenced the final proportions of type I and II chondrules, hence the bulk redox state of a chondrite. Or the mineralogical diversity and complexity of a matrix is probably greater when its condensed precursors are more diverse and out of equilibrium.

References

[1] Yoneda, S. & Grossman, L. Condensation of CaO-MgO-Al_2O_3-SiO_2 liquids from cosmic gases. Geochim. Cosmochim. Acta **59** (16), 3413–3444 (1995). https://doi.org/10.1016/0016-7037(95)00214-K .

[2] Davis, A. & Richter, F. Condensation and evaporation of solar system materials. *Treatise on Geochemistry* **1**, 711 (2003) .

[3] Palme, H., Lodders, K. & Jones, A. Solar system abundances of the elements. *Planets, Asteriods, Comets and The Solar System, Volume 2 of Treatise on Geochemistry (Second Edition). Edited by Andrew M. Davis. Elsevier, 2014., p. 15-36* **2** (2014) .

[4] Pignatale, F. C., Charnoz, S., Chaussidon, M. & Jacquet, E. Making the Planetary Material Diversity during the Early Assembling of the Solar

System. ApJ **867** (2), L23 (2018). https://doi.org/10.3847/2041-8213/aaeb22, arXiv:1810.10827 [astro-ph.EP].

[5] Morbidelli, A., Libourel, G., Palme, H., Jacobson, S. A. & Rubie, D. C. Subsolar Al/Si and Mg/Si ratios of non-carbonaceous chondrites reveal planetesimal formation during early condensation in the protoplanetary disk. *Earth and Planetary Science Letters* **538**, 116220 (2020). https://doi.org/10.1016/j.epsl.2020.116220, arXiv:2003.05486 [astro-ph.EP].

[6] Kitzmann, D., Stock, J. W. & Patzer, A. B. C. FASTCHEM COND: equilibrium chemistry with condensation and rainout for cool planetary and stellar environments. MNRAS **527** (3), 7263–7283 (2024). https://doi.org/10.1093/mnras/stad3515, arXiv:2309.02337 [astro-ph.EP].

[7] Grossman, L. Condensation in the primitive solar nebula. *Geochimica et Cosmochimica Acta* **36** (5), 597–619 (1972) .

[8] Ebel, D. S., Lauretta, D. & McSween, H. Condensation of rocky material in astrophysical environments. *Meteorites and the early solar system II* **1**, 253–277 (2006) .

[9] Petaev, M. I. & Wood, J. A. The condensation with partial isolation model of condensation in the solar nebula. *Metoritics and Planetary Science* **33** (5), 1123–1137 (1998). https://doi.org/10.1111/j.1945-5100.1998.tb01717.x .

[10] Gordon, S. & McBride, B. J. Computer Program For Calculation Of Complex Chemical Equilibrium Compositions And Applications Ii. Users Manual And Program Description. *NASA reference publication 1311* (1996) .

[11] Nagahara, H. & Ozawa, K. Evaporation of forsterite in h2 gas. *Geochimica et Cosmochimica Acta* **60** (8), 1445–1459 (1996). URL https://www.sciencedirect.com/science/article/pii/0016703796000142. https://doi.org/https://doi.org/10.1016/0016-7037(96)00014-2 .

[12] Tsuchiyama, A., Takahashi, T. & S., T. Evaporation rates of forsterite in the system Mg2SiO4 and H2. *Mineralogical Journal* **20** (3), 113–126 (1998). https://doi.org/10.2465/minerj.20.113 .

[13] Richter, F. M., Davis, A. M., Ebel, D. S. & Hashimoto, A. Elemental and isotopic fractionation of Type B calcium-, aluminum-rich inclusions: experiments, theoretical considerations, and constraints on their thermal evolution. *GCA* **66** (3), 521–540 (2002). https://doi.org/10.1016/S0016-7037(01)00782-7 .

[14] Gurvich, I., Lev Veniaminovich; Veyts & C.B., A. *Thermodynamic properties of individual substances: elements and compounds* Vol. 2 (Hemisphere Pub. Corp., New York, NY., 1991).

[15] Chase, M. *Thermodynamic properties of individual substances* (Begell House, New York, 1996).

[16] Berman, R. *PHD Thesis* (University of British Columbia, 1983).

[17] Berman, R. G. & Brown, T. H. Heat capacity of minerals in the system Na_2O-K_2O-CaO-MgO-FeO-Fe_2O_3-Al_2O_3-SiO_2-TiO_2-H_2O-CO_2: representation, estimation, and high temperature extrapolation. *Contributions to Mineralogy and Petrology* **89** (2-3), 168–183 (1985). https://doi.org/10.1007/BF00379451 .

[18] Berman, R. G. Internally-Consistent Thermodynamic Data for Minerals in the System Na2O-K2O-CaO-MgO-FeO-Fe2O3-Al2O3-SiO2-TiO2-H2O-CO2. *Journal of Petrology* **29** (2), 445–522 (1988). https://doi.org/10.1093/petrology/29.2.445 .

[19] Zimmer, K. *et al.* Supcrtbl: A revised and extended thermodynamic dataset and software package of supcrt92. *Computers & geosciences* **90**, 97–111 (2016) .

[20] Chase, M. *NIST-JANAF Thermochemical Tables, 4th Edition* (American Institute of Physics, -1, 1998).

[21] Pankratz, L. & Mrazek, R. V. Thermodynamic properties of elements and oxides. *Bur. Mines Bul.* (672) (1983) .

[22] Pankratz, L., Mah, A. D. & Watson, S. *Thermodynamic properties of sulfides* 689 (US Department of the Interior, Bureau of Mines, 1987).

[23] Birnstiel, T., Ormel, C. W. & Dullemond, C. P. Dust size distributions in coagulation/fragmentation equilibrium: numerical solutions and analytical fits. A&A **525**, A11 (2011). https://doi.org/10.1051/0004-6361/201015228, arXiv:1009.3011 [astro-ph.EP].

[24] Drażkowska, J. & Dullemond, C. P. Can dust coagulation trigger streaming instability? A&A **572**, A78 (2014). https://doi.org/10.1051/0004-6361/201424809, arXiv:1410.3832 [astro-ph.EP].

[25] Petaev, M. I., Wood, J. A., Meibom, A., Krot, A. N. & Keil, K. The ZONMET thermodynamic and kinetic model of metal condensation. Geochim. Cosmochim. Acta **67** (9), 1737–1751 (2003). https://doi.org/10.1016/S0016-7037(02)00956-0 .

[26] Birnstiel, T., Klahr, H. & Ercolano, B. A simple model for the evolution of the dust population in protoplanetary disks. A&A **539**, A148 (2012). https://doi.org/10.1051/0004-6361/201118136, arXiv:1201.5781 [astro-ph.EP].

[27] Petaev, M. The GRAINS thermodynamic and kinetic code for modeling nebular condensation. *CALPHAD* **33**, 317–327 (2003). https://doi.org/10.1016/j.calphad.2008.12.001 .

[28] Woitke, P. *et al.* Equilibrium chemistry down to 100 K. Impact of silicates and phyllosilicates on the carbon to oxygen ratio. A&A **614**, A1 (2018). https://doi.org/10.1051/0004-6361/201732193, arXiv:1712.01010 [astro-ph.EP].

[29] Woitke, P. *et al.* Equilibrium chemistry down to 100 K. Impact of silicates and phyllosilicates on the carbon to oxygen ratio. A&A **614**, A1 (2018). https://doi.org/10.1051/0004-6361/201732193, arXiv:1712.01010 [astro-ph.EP].

[30] Fegley, J., Bruce. Nuth, J. A. & Sylvester, P. (eds) *Cosmochemical Trends of Volatile Elements in the Solar System.* (eds Nuth, J. A. & Sylvester, P.) *Origins of Solar Systems*, 51 (1988).

[31] Hong, Y. & Fegley Jr, B. Experimental studies of magnetite formation in the solar nebula. *Meteoritics & Planetary Science* **33** (5), 1101–1112 (1998) .

[32] Lauretta, D. S., Lodders, K. & Fegley Jr, B. Experimental simulations of sulfide formation in the solar nebula. *Science* **277** (5324), 358–360 (1997) .

[33] Fegley, J., Bruce. Kinetics of gas-Grain Reactions in the Solar Nebula. Space Sci. Rev. **92**, 177–200 (2000). https://doi.org/10.1023/A:1005286910756 .

[34] Nagahara, H. Kinetics of gas–solid reactions in the solar system and beyond. *High Temperature Gas-Solid Reactions in Earth and Planetary Processes* 461–498 (2018) .

[35] Imae, N., Tsuchiyama, A. & Kitamura, M. An experimental study of enstatite formation reaction between forsterite and si-rich gas. *Earth and planetary science letters* **118** (1-4), 21–30 (1993) .

[36] Unterborn, C. T. & Panero, W. R. The Effects of Mg/Si on the Exoplanetary Refractory Oxygen Budget. ApJ **845** (1), 61 (2017). https://doi.org/10.3847/1538-4357/aa7f79, arXiv:1604.08309 [astro-ph.EP].

[37] Greshake, A. The primitive matrix components of the unique carbonaceous chondrite ACFER 094: A TEM study. Geochim. Cosmochim. Acta **61** (2), 437–452 (1997). https://doi.org/10.1016/S0016-7037(96)00332-8 .

[38] Desch, S. J. & Connolly, H. C., Jr. A model of the thermal processing of particles in solar nebula shocks: Application to the cooling rates of chondrules. *Meteoritics and Space Science* **37** (2), 183–207 (2002). https://doi.org/10.1111/j.1945-5100.2002.tb01104.x .

[39] Ciesla, F. J., Hood, L. L. & Weidenschilling, S. J. Evaluating planetesimal bow shocks as sites for chondrule formation. *Meteoritics and Space Science* **39** (11), 1809–1821 (2004). https://doi.org/10.1111/j.1945-5100.2004.tb00077.x .

Acknowledgments

We thank the anonymous reviewers for their comments that improved the quality of the paper. Parts of this work were supported by the DISKBUILD project (ANR-20- CE49-0006), the LabEx UnivEarthS initiative (ANR-10-LABX-0023 and ANR-18-IDEX-0001) and by the French space agency CNES (Centre National d'Études Spatiales). The numerical calculations were performed in part on the S-CAPAD/DANTE platform at IPGP. MC acknowledges funding from the ERC under the Horizon Europe program/ERC grant agreement No. 101200693 (DUST). This work was partially supported by ANR PERSEID (ANR-25-CE49-3880, PI: Yves Marrocchi). PAS was supported by the Swiss National Science Foundation (SNSF) through an Eccellenza Professorship (203668) and the Swiss State Secretariat for Education, Research and Innovation (SERI) under contract No. MB22.00033, a SERI-funded ERC Starting grant "2ATMO". SC thanks G. Avice, J. Siebert and F. Moynier for fruitful discussions.

Competing interests

The authors declare no competing interests.

Authors contributions

S. Charnoz has designed and led the project; conceived, built and tested `KineCond`. All authors participated equally in the writing. J. Aléon provided his expertise on CAIs, mineral condensation processes and helped design and testing `KineCond`. M. Chaussidon provided his expertise on CAIs and chondrites physics; Y. Marrocchi provided his expertise on chondrites, AOA, and chondrules. P. Sossi provided his expertise and chondrites and thermodynamics and performed the fO_2 equilibrium calculations using the `factsage` software. P. Franco performed equilibrium calculations with FASTCHEM-COND.

Additional information

High resolution mosaic images of kinetic condensation sequences displayed in poster format (A0) are available to download from the IPGP Research Collection repository with DOI number : *doi.org/10.18715/IPGP.2026.mkv2scjh*. The link to the repository is https://doi.org/10.18715/IPGP.2026.mkv2scjh .

Data availability

The data used to produce Figures 1 to 3 were generated by the `KineCond` code, available in the public repository : IPGP research Collection https://doi.org/10.18715/IPGP.2026.mkv2scjh.

Code availability

A version of the `KineCond` code, configurated to reproduce the main results of this paper is available in the public repository : IPGP research Collection https://doi.org/10.18715/IPGP.2026.mkv2scjh.
